# Computational investigation of the impact of core sequence on immobile DNA four-way junction structure and dynamics

**DOI:** 10.1093/nar/gkab1246

**Published:** 2021-12-22

**Authors:** Matthew R Adendorff, Guo Qing Tang, David P Millar, Mark Bathe, William P Bricker

**Affiliations:** Department of Biological Engineering, Massachusetts Institute of Technology, Cambridge, MA 02139, USA; Department of Molecular Biology, Scripps Research Institute, La Jolla, CA 92037, USA; Department of Integrative Structural and Computational Biology, Scripps Research Institute, La Jolla, CA 92037, USA; Department of Biological Engineering, Massachusetts Institute of Technology, Cambridge, MA 02139, USA; Department of Biological Engineering, Massachusetts Institute of Technology, Cambridge, MA 02139, USA; Department of Chemical and Biological Engineering, University of New Mexico, Albuquerque, NM 87131, USA

## Abstract

Immobile four-way junctions (4WJs) are core structural motifs employed in the design of programmed DNA assemblies. Understanding the impact of sequence on their equilibrium structure and flexibility is important to informing the design of complex DNA architectures. While core junction sequence is known to impact the preferences for the two possible isomeric states that junctions reside in, previous investigations have not quantified these preferences based on molecular-level interactions. Here, we use all-atom molecular dynamics simulations to investigate base-pair level structure and dynamics of four-way junctions, using the canonical Seeman J1 junction as a reference. Comparison of J1 with equivalent single-crossover topologies and isolated nicked duplexes reveal conformational impact of the double-crossover motif. We additionally contrast J1 with a second junction core sequence termed J24, with equal thermodynamic preference for each isomeric configuration. Analyses of the base-pair degrees of freedom for each system, free energy calculations, and reduced-coordinate sampling of the 4WJ isomers reveal the significant impact base sequence has on local structure, isomer bias, and global junction dynamics.

## INTRODUCTION

Nucleic acid nanotechnology offers a powerful synthetic approach to program static (1–13) and dynamic (14–16) molecular constructs via self-assembly. These assemblies have been used in a variety of applications, including biomimicry (17), programmed energy transfer and photonics (18–22), drug delivery (23,24), actuated nanoreactor design (25) and molecular sensing (26). While the ability to program nucleic acid sequence from the bottom-up offers the opportunity to rationally control DNA nanostructure shape and dynamics, our understanding of the structure and conformational dynamics of elemental building blocks of nucleic acid nanostructures remains limited. For example, the relative roles of 4WJ isomeric state preferences (27–29), single- versus double-crossover (30–32), and nicks (33–35) on DNA construct equilibrium structure and dynamics, together with their sequence-dependence, remain poorly understood.

**Figure 1.**
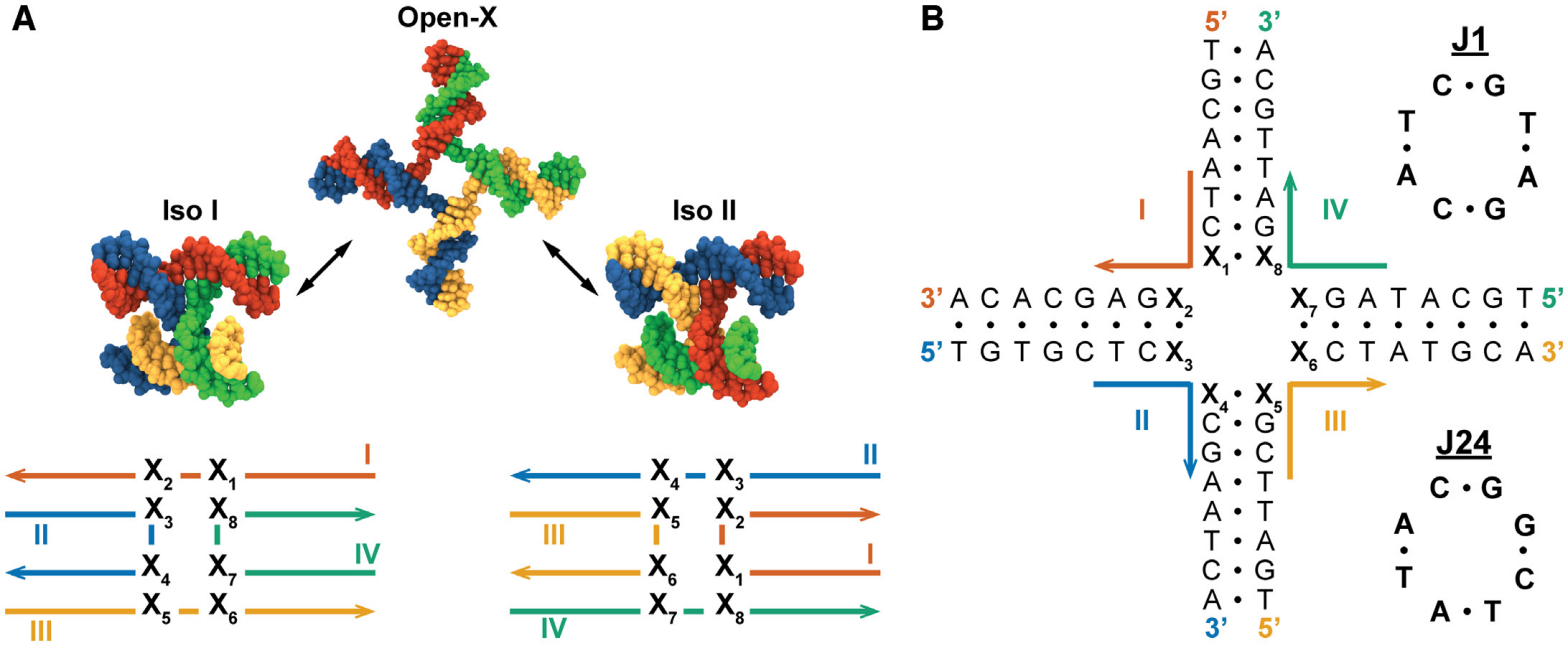
Modeling DNA 4WJ isomers. (**A**) The three major isomers of DNA 4WJs. Switching between the Iso I and Iso II junction forms alters the base stacking at the site of strand crossover, with the X3/X8 and X7/X4 pairs being replaced with X5/X2 and X1/X6. (**B**) The base-pair sequence of the junction systems used in this study, shown in the Open-X configuration. Core base-pair sequences, denoted X_1−8_, are assigned to sequences of J1 (top) and J24 (bottom), which are canonical Seeman 4WJ designs.

The immobile 4WJ is the core structural component of DNA nanotechnology based on Seeman’s foundational work (1,36). The three-dimensional structure of these DNA junctions in isolation has been extensively studied experimentally (37–39). In isolation the 4WJ exists in one of three global conformations, including two stacked antiparallel junction isomers and an unstacked open-X conformation, depending on ionic conditions (Figure 1A). These stacked forms are adopted in the presence of polyvalent ions or in a high monovalent salt environment (40–42) due to the screening of phosphate charges at the junction center, which renders the stacked-X conformation unstable with respect to the open-X form when not in an ionic environment (43). The stacked-X conformation of a specific asymmetric junction has been shown to have a right-handed *J*_twist_ angle of close to 60°, compared to a shallower *J*_twist_ angle seen in symmetric junctions (44). In addition, the relative populations of these stacked conformational isomers have been shown to be sensitive to Mg^2 +^ concentration (45) and to the base-pair sequence at the site of strand crossover (40,46,47), as well as at the first (27,48) and second nearest-neighbour base-pairs (28,29). The conformational heterogeneity of the junction has also been shown to be sequence dependent through characterization of different junction core arrangements by NMR spectroscopy and time-resolved Förster resonance energy transfer (tr-FRET) (27).

While the primary topological difference between these isomers is a change in base-pair stacking at the junction core, differences in duplex stacking energies (34,35,39) are insufficient to fully account for the experimentally observed isomer ratios (38,39). A contribution of the geometric constraints imposed at the junction core, in the stacked isomeric forms, to the isomerization free energy difference has also been considered (37); and the effect of a sequence-dependent electrostatic potential on junction stacking preferences has been suggested by selective base-charge neutralization (49). However, a detailed understanding of the topological and energetic contributions to junction isomerization bias is still lacking. Further, the impact of these contributions on junction flexibility remains unknown. Understanding the sequence-level origins of these features may offer generalizable design principles that may eventually be used to inform DNA nanostructure design.

The application of all-atom molecular dynamics (MD) to nucleic acids has offered fundamental insights into the sequence-dependent structure and deformability of canonical B-form duplexes (50–58), Watson-Crick base-pairing energies (59), solvent effects on the stabilities of A-form and B-form duplexes (60), the free-energies of base-pair mismatches (61), and the conformational dynamics of larger-scale origami objects (11,12,62–65). In addition, coarse-grained MD (66) is often utilized for simulation of larger-scale nucleic acid nanostructures, and accurate sequence-level conformational data is of particular importance to parameterize these methods. All-atom MD has also been used to investigate several junction systems, illustrating the structure of stacked isomers (62), the ionic environment around these systems (67), protein-junction interactions (68), and suggesting reaction coordinates for the isomerization process in terms of simple collective variables (69,70). These investigations focused on junctions containing inverted repeat sequences, mismatched bases, or mobile junction core sequences, however; and no dedicated analysis of sequence-level structure and energetics in isolated asymmetrical and immobile junction isomers has been performed. In addition, a recent paper details some of the difficulty with characterizing precise interactions in closely-packed DNA structures, including an overestimation of binding affinities of cationic species to phosphate groups and inter-strand interactions that are too strong (71).

In this work, we use all-atom MD to simulate multiple replicates of both stacked isomers for the Seeman J1 sequence and for a variant sequence, J24 (Figure 1B), which possesses the same GC content as J1 but differs in its core base stacking arrangement. Calculations of the base-pair level structure are performed on the resulting trajectories using established helical parameter analysis methods and the distributions of these parameters, as well as their inherent flexibilities, are compared to reference B-form duplex, nicked-duplex and single crossover topological variants of the J1 and J24 junctions. The per-base free energies of isomerization are then determined from structural ensembles of each junction sequence using end-state free energy calculations. Free energies of isomerization from the simulated MD ensembles are additionally compared to experimental isomer ratios from tr-FRET measurements. Finally, the essential dynamics of 4WJs are derived from meta-ensemble principal component analysis (PCA) of the junction trajectories and the potentials of mean force (PMFs) along the principal junction twist coordinate are calculated using enhanced sampling with a biasing potential.

## MATERIALS AND METHODS

### Junction nomenclature

**Figure 2.**
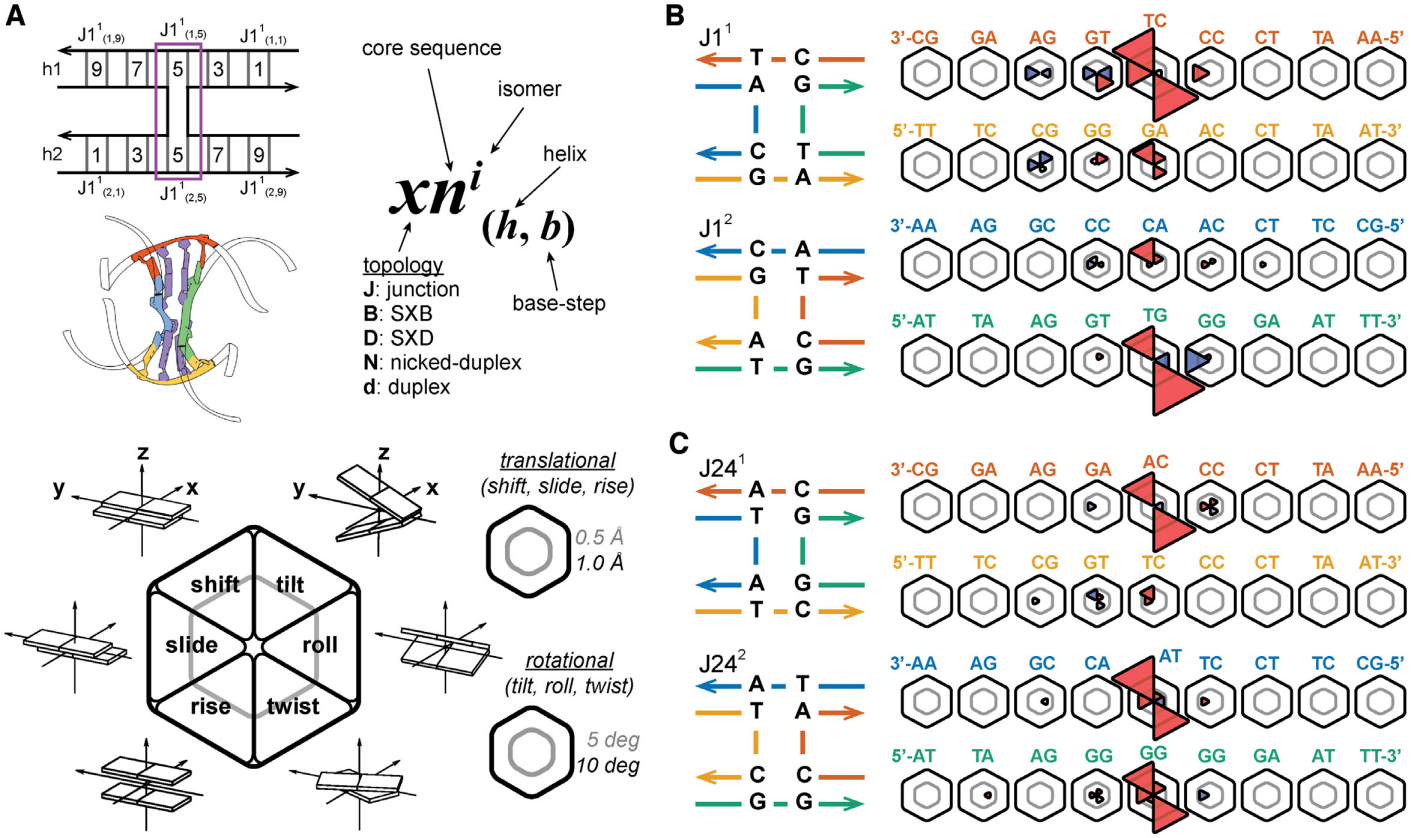
The DNA pseudo-duplexes within 4WJs can deviate significantly from canonical B-form structure. (**A**) Six helical step parameters (72,73) are calculated for each base-pair step comprising the two junction helix arms (h1 and h2). Using the J1 4WJ as an example (top, left), the notation, $xn^i_{(h,b)}$, used throughout this paper denotes the topology, core sequence, isomer, helix, and base-step (top, right). An atomic representation is shown (middle, left) with step 5 of both helices shown in purple. Deviations in mean parameter values from reference B-form simulations (duplex) for each step are shown as wedges in a hexagon (representative plot at lower left). The inner gray line represents a deviation of 0.5 Å for translational parameters (shift, slide, and rise) and a deviation of 5° for rotational parameters (tilt, roll, and twist); the outer black line represents deviations of 1.0 Å and 10° respectively. Negative deviations are coloured red, positive deviations are coloured blue, and the absence of a wedge indicates a deviation of less than 0.2 Å or 2°. Within the base-pair step coordinate frame, the x-axis points away from the minor groove edge of a base-pair, the y-axis points towards the complementary base, and the z-axis points in the direction of helical rise (72,73). (**B**) Deviations in step parameter mean values for the J1 junction systems (right) and their topologies (left). Isomer 1 is shown on top (first two helices) with isomer 2 below (third and fourth helices) for all systems. Base-pair step sequences are shown for strands I and III (red, orange) for isomer 1; and strands II and IV (blue, green) for isomer 2; the 5′ to 3′ step sequence is read right to left for helix 1 and left to right for helix 2 for all systems. (**C**) Deviations in step parameter mean values for the J24 junction systems (right) and their topologies (left).

**Figure 3.**
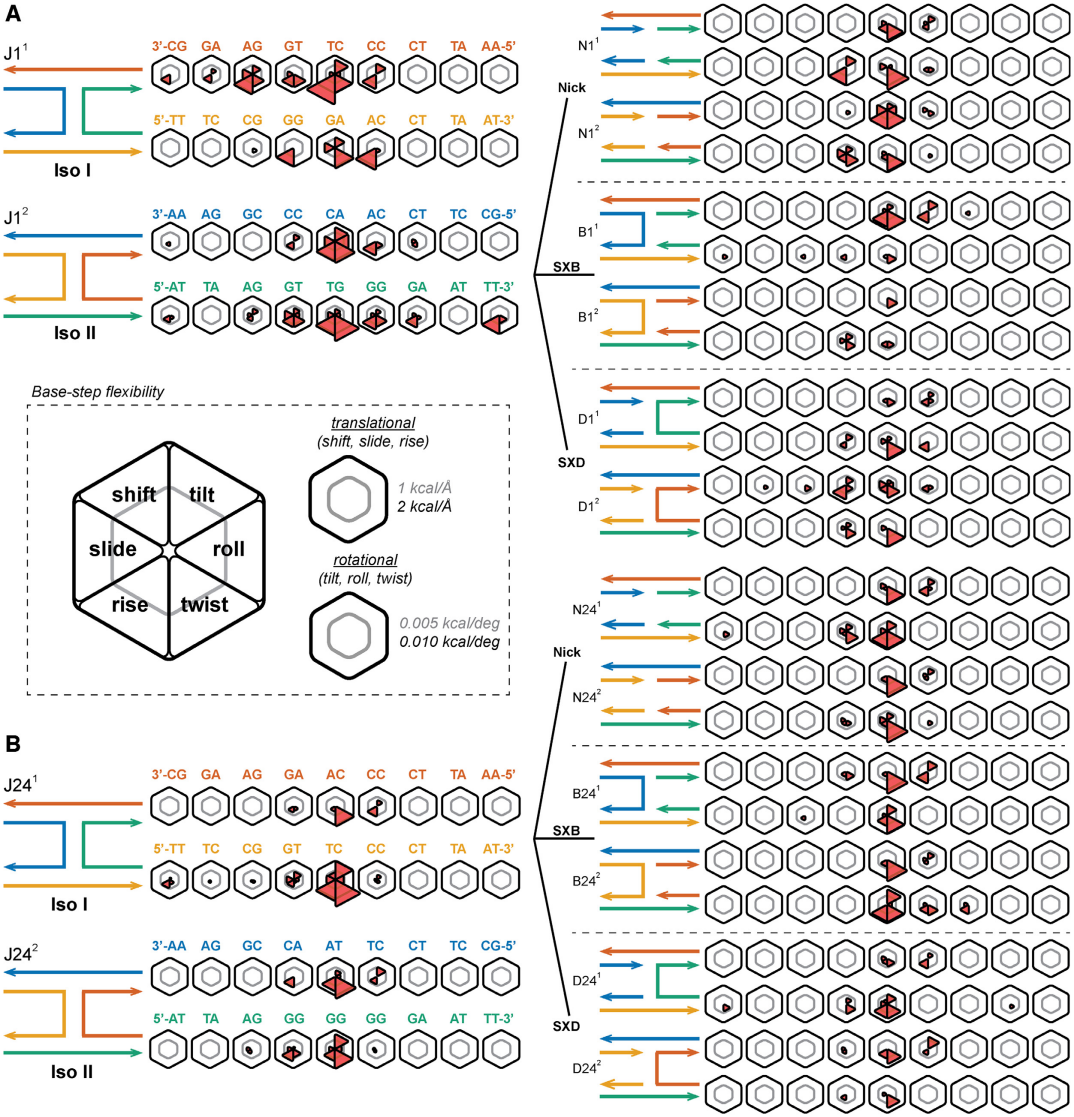
The effect of junction topology on base-pair step flexibility. (**A**) Deviations in the helical step parameter stiffness constants from those of canonical B-form DNA are shown for the J1 junction systems (left), the nicked duplex systems (top right), the SXB junction systems (middle right), and the SXD junction systems (bottom right). Base-pair step sequences are shown for strands A (red in isomer 1, blue in isomer 2) and C (orange in isomer 1, green in isomer 2); the 5 to 3 sequence runs right to left for helix 1 and left to right for helix 2 for both isomers. Topology schematics for J1^1^ and J1^2^ are shown top left and bottom left respectively. Complementary schematics for the nick, SXB and SXD systems are shown top right, middle right, and bottom right respectively. (**B**) In comparison, deviations in the helical step parameter stiffness constants from those of canonical B-form DNA are shown for the J24 junction systems (left), the nicked duplex systems (top right), the SXB junction systems (middle right), and the SXD junction systems (bottom right). (center inset) The inner gray line in each hexagon represents a deviation of 1 kcal/Å for the translational parameters (shift, slide and rise) and of 0.005 kcal/deg for the rotational parameters (tilt, roll and twist); the outer black line represents deviations of 2 kcal/Å and 0.01 kcal/deg respectively.

A shorthand notation is introduced in this work for referencing junction core sequence, isomeric state, helical arm and base-pair step identities. A particular base-pair step (Figure 2A), defined between two adjacent base-pairs, is denoted using the form $xn^i_{(h,b)}$, where *x* is the junction topology (J: junction, B: SXB, D: SXD, N: nicked-duplex, and d: duplex), *n* is the core sequence, i.e. 1 or 24 in this work, *i* is the isomer number (1 or 2), *h* is the helix number for the pseudo-duplexes (1 or 2) and *b* is the base-pair step index (1 to 9, leaving out the terminal three base-pair steps). The helices are defined such that helix 1 includes strand I in isomer 1 and strand II in isomer 2; helix 2 includes strand III in isomer 1 and strand IV in isomer 2. The base-pair step sequence and numbering follows the 5′ to 3′ sequence of strand I in isomer 1 helix 1, strand III in isomer 1 helix 2, strand II in isomer 2 helix 1, and strand IV in isomer 2 helix 2. The terminal three base-pair steps on either end of the helical arms are not included in helical parameter analyses and so the numbering starts at the fourth base-pair step in the helix and runs up to and includes the twelfth step. These terminal base-pair steps are excluded due to the common occurrence of fraying effects at terminal base-pairs. Several topological variants of the J1 and J24 junction systems are also investigated in this work. The first two of these variants are the single-crossovers, motifs that only have one crossing strand at the junction site. When the crossing occurs on the left-hand side of the junction frame shown in Figure 3, the variant is termed the SXB construct and if the crossing occurs on the right it is termed the SXD construct, for a given isomer. The junction nomenclature used for indexing is as previously noted, e.g. the single-crossover equivalent of J1^1^, with strand II performing the crossing action, is denoted B1^1^; and the equivalent of J1^2^, with strand I performing the crossing action, is D1^2^. The second type of variant is the nicked-duplex, which is a B-form duplex with the same base-pair sequence as a junction arm pseudo-duplex but with a phosphate backbone break at the junction crossover site, e.g. between X3 and X8 in J1$_{(1)}^1$. As noted above, the nicked-duplex equivalent of J1$_{(1)}^2$ is written as N1$_{(1)}^2$. The last variant is the duplex, identical to the nicked-duplex without the phosphate backbone break. The duplex equivalent of J1$_{(1)}^2$ is written as d1$_{(1)}^2$ (note the lower-case *d* to differentiate from the SXD construct).

### Construction of all-atom systems

The junction strand sequences used were identical to previous experimental systems used for J1 (36) and J2 (27), up to and including the fifth neighbour from the junction core. All-atom PDB files for J1^1^, J1^2^, J24^1^ and J24^2^ were generated using the atomic structure generator in the lattice-free implementation of CanDo (32,65), with a starting *J*_*twist*_ (74) value of 60° (right-handed junction). The system was immersed in TIP3P water (75), then explicit Na^+^, Mg^2 +^ and Cl^−^ ions were added to neutralize DNA backbone charges and to set the simulation cell Na^+^, Mg^2 +^ ion concentrations to 50 and 5 mM, respectively; consistent with the aforementioned experimental conditions. The duplex, nicked-duplex and single crossover (B-strand and D-strand) equivalents of the J1 and J24 junction isomers were generated from the initial full junction structures using Discovery Studio Visualizer (Version 4.5; Dassault Systémes) and then were solvated and ionized in the same manner as the junction systems.

### Molecular dynamics simulations

All simulations were performed using the program NAMD2 (76) with the CHARMM27 force field (77,78) and Allnér Mg^2 +^ (79) parameters. This procedure as follows has been successfully utilized in several previous studies of DNA origami nanostructures (80,81). An integration time step of 2 fs and periodic boundary conditions were applied in an orthogonal simulation cell. Van der Waals energies were calculated using a 1.2 nm cut-off with a switching function applied from 1.0 to 1.2 nm and the pair list distance at 1.4 nm. The Particle Mesh Ewald (PME) method (65) was used to calculate full electrostatics with a maximum grid point spacing of 0.1 nm. Full electrostatic forces were computed every two time steps (every 4 fs) and non-bonded forces were calculated at each time step (2 fs). Simulations were performed in the *NpT* ensemble using the Nosé-Hoover Langevin piston method (82,83) for pressure control with an oscillation period of 200 fs and a damping time of 100 fs. Langevin forces (84) were applied to all heavy atoms for temperature control (298 K) with coupling coefficients of 5 ps^−1^. All hydrogens were constrained to their equilibrium lengths during the simulation and system configurations were recorded every 1 ps for downstream analysis of coordinates. Energy minimization was always performed on the orthogonal simulation cell prior to dynamics using the conjugate-gradient and line search minimizer implemented in NAMD2, first on the solvent and ions alone for 10,000 steps with all nucleic acid atoms spatially constrained, followed by an additional 10,000 steps with all atoms unconstrained. All parameters for the minimization were identical to those used for dynamics. The system was then slowly heated (1 K per 10 ps) to 298 K and the pressure was allowed to equilibrate to 1 atm prior to the production run MD. All simulated systems (whole junction and duplex, nicked-duplex, and single crossover equivalents) were run for 300 ns, in triplicate (900 ns total production time per system). The first 60 ns of each simulation was considered as equilibration time and was not used for subsequent analysis. It should be noted that a recent study details several issues with MD simulations of Holliday junctions undergoing unexpected conformational transitions, although the present study utilizes a different force field, Holliday junction sequences, and conditions to this paper (71).

### Base-pair parameter analysis

DNA base-pair parameters were calculated at 10 ps intervals for all trajectories using the 3DNA program (72,73) with the MDAnalysis x3DNA interface (85). The stiffness matrix in base-pair step helicoidal space (rise, shift, slide, roll, tilt, twist) was calculated from the simulation-obtained helicoidal covariance matrix, **C**_*h*_, as:

(1) $$\begin{equation*} {\bf C}_h = k_B T {\bf F}_h^{-1} \end{equation*}$$

where **F**_*h*_ is the helicoidal stiffness matrix, ${\bf F}_h^{-1}$ is its inverse, *k*_*B*_ is Boltzmann’s constant and *T* is the temperature. The determinant of **C**_h_ (analogous, in this case, to the product of the eigenvalues of the matrix) was used to calculate the conformational volume of a base-pair step which takes into account the off-diagonal stiffness terms in a general description of base-pair step flexibility (50,51).

### MM-PBSA free energy analysis

The end-state MM-PBSA method (60) was used to estimate the free energy of the J1 and J24 isomer systems using the following equations:

(2) $$\begin{equation*} \Delta G_{solvated} = E_{gas} + \Delta G_{solvation} - TS_{solute} \end{equation*}$$

(3) $$\begin{equation*} E_{gas} = E_{internal} + E_{electrostatic} + E_{vdW} \end{equation*}$$

(4) $$\begin{equation*} \Delta G_{solvation} = \Delta G_{PB} + \Delta G_{SA} \end{equation*}$$

where *E*_*gas*_ is the gas phase energy of a molecule’s conformation as described by the mechanical force field, Δ*G*_*solvation*_ is the solvation free energy, *T* is the temperature, and *S*_*solute*_ is the solute entropy. *E*_*gas*_ comprises the internal energy (*E*_*internal*_), i.e. contributions from bond, angle, and dihedral energies, and energies due to non-bonded electrostatic (*E*_*electrostatic*_) and van der Waal’s (*E*_*vdW*_) interactions. Δ*G*_*solvation*_ comprises the polar solvation (Δ*G*_*PB*_) and the non-polar solvation (Δ*G*_*SA*_) free energies.

The MMPBSA.py module (86) of AmberTools14 (87) was used to run all calculations with CHAMBER-generated topologies (88). 24,000 frames (10 ps between frames) were used from each trajectory to generate the energy averages with 72 000 frames analysed per isomer (three total trajectories per isomer). Gas phase energies (*E*_*gas*_) were calculated from the CHARMM27 force field with 1–4 non-bonded interaction energies summed into their corresponding van der Waal’s and electrostatic terms.

Both solvation terms (Δ*G*_*PB*_ and Δ*G*_*SA*_) were calculated using the pbsa module in sander, both included in AmberTools14. The electrostatic field, including the solvent reaction field and the Coulombic field, was described with the Poisson-Boltzmann equation (PBE) (89,90):

(5) $$\begin{eqnarray*} \nabla \cdot (\varepsilon (r) \nabla \phi (r)) &=& -4 \pi \rho (r) - 4 \pi \lambda (r) \sum _i z_i c_i \nonumber \\ &&\times\ \exp \left( \frac{-z_i \phi (r)}{k_B T} \right) \end{eqnarray*}$$

where ϵ(*r*) is the dielectric constant, ϕ(*r*) is the electrostatic potential, ρ(*r*) is the solute charge, λ(*r*) is the ion exclusion or Stern layer masking function describing ion accessibility of position *r*, $z$_*i*_ is the charge of ion type *i*, *c*_*i*_ is the bulk number density of ion type *i* far from the solute, *k*_*B*_ is the Boltzmann constant, and *T* is the temperature; the summation is over all ion types. The non-linear PBE (91) was solved on a 0.5 Å spacing finite difference grid with 5000 iterations per frame. The solute and solvent dielectric constants were set to 1.0 and 80.0, respectively, with the weighted harmonic average level set function being used for boundary grid edges. The solvent excluded surface (92) was used to define the dielectric boundary and was generated using a 1.4 Å solvent probe radius and CHARMM atomic radii. Per-base decomposition was performed in AmberTools to obtain the interaction energies of each base with all other bases. Decomposition of the per-base surface area contributions was performed using the Linear Combination of Pairwise Overlaps (LCPO) method (93). Full molecule surface area contributions were calculated within the pbsa framework.

The solute entropy was approximated from the covariance matrix obtained in each simulation using Schlitter’s method (94), where the mass-weighted covariance matrix **C**′ is determined from the covariance matrix **C** and the mass matrix **M**:

(6) $$\begin{equation*} {\bf C}^{\prime } = {\bf M}^{1/2} {\bf C}^{\prime } {\bf M}^{1/2} \end{equation*}$$

The matrix **M** contains the atomic masses along the diagonal and is zero elsewhere. Diagonalization of **C**′ by an orthogonal transformation returns the classical variances of the new coordinates *q*_*i*_ whose fluctuations are linearly uncorrelated. The entropy is then estimated as a sum of the contributions (*S*_*i*_) from all individual coordinate components:

(7) $$\begin{equation*} S < S^{\prime } = \frac{1}{2} k \sum ln \left[ 1 + \left( k T e^2 / \hbar ^2 \right) \langle q_i^2 \rangle \right] \end{equation*}$$

where the calculated estimate *S*′ is an upper bound for the real entropy *S*.

### Essential dynamics analysis

An ensemble of junction coordinates obtained from MD simulation was subjected to principal component analysis to extract the essential modes (54,95), the coordinate fluctuations responsible for most of the variance observed in the system. A meta-ensemble (96) was generated from the backbone atoms of all four isomer simulation sets comprising 2.88 μs of total dynamics time. The terminal three base-pairs at both the 5′ and 3′ ends of the junction arm pseudo-duplexes were excluded from this ensemble to avoid the influence of end-effects including fraying.

The average structural coordinate set for a given ensemble was generated through iterative superposition of all trajectory frames. Initially, all frames were superposed onto the first trajectory frame, followed by calculation of the mean coordinates and the setting of these as the new reference set. This process was repeated until the total RMSD was lower than 0.0001 Å. Once all frames had been aligned to the average structure, the covariance matrix **C** was constructed:

(8) $$\begin{equation*} {\bf C} = \langle (x(t) - \langle x \rangle ) (x(t) - \langle x \rangle )^{\bf T} \rangle \end{equation*}$$

where 〈 · 〉 denotes the ensemble average and **C** is a symmetric matrix. Diagonalization of **C** was then performed by an orthogonal transformation **T**:

(9) $$\begin{equation*} {\bf C} = {\bf T} \Lambda {\bf T}^{\bf T} \end{equation*}$$

where Λ is the eigenvalue matrix and **T** contains the eigenvectors of **C**. The eigenvalues (λ) relate to the mean square fluctuations (in Å^2^) along the eigenvector coordinates and represent the contribution of each component to the total system fluctuation. The six zero eigenvalues, corresponding to global rotation and translation (removed through the superposition step), and their eigenvectors were discarded and the remaining vectors were sorted in descending order by their eigenvalues (Supplementary Figure S43).

The original molecular configurations were projected onto the first three principal components to yield their principal coordinates *p*_*i*_(*t*):

(10) $$\begin{equation*} p_i(t) = \nu _i \cdot (x(t) - \langle x \rangle ) \end{equation*}$$

where ν_*i*_ is the *i*th eigenvector of *C* and $\langle p_i^2 \rangle = \lambda _i$. For visualization purposes, projections along eigenvectors were transformed back to Cartesian coordinates with:

(11) $$\begin{equation*} x_i^*(t) = p_i(t) \cdot \nu _i + \langle x \rangle \end{equation*}$$

All essential mode analyses were performed with the ProDy package (97).

### Enhanced sampling with the adaptive biasing force method

The free energy profile, *A*(ξ), along the slowest essential mode of the junction backbone meta-ensemble was calculated as a function of the collective variable (colvar) ξ, here representing *J*_*twist*_; defined as the projection of a junction system’s backbone atom deviations from the meta-ensemble’s average structure coordinates onto a vector in *R*^3*n*^, where *n* is the number of atoms. The computed quantity is:

(12) $$\begin{eqnarray*} && p \left( \lbrace x_i(t)\rbrace ,\lbrace x_i^{ref}\rbrace \right) = \sum _{i=1}^n \nu _i \nonumber \\ &&\quad \times\, \left( U(x_i(t) - x_{cog}(t) - (x_i^{ref} - x_{cog}^{ref}) \right) \end{eqnarray*}$$

where *U* is the optimal rotation matrix, *x*_*cog*_(*t*) and $x_{cog}^{ref}$ are the centers of geometry of the current and reference positions and ν_*i*_ are the per-atom components of the vector (98). *A*(ξ) was obtained using the NAMD2.10 implementation of the Adaptive Biasing Force (ABF) method of thermal integration (99,100). The algorithm comprises two steps: (1) the thermodynamic force acting along ξ is extracted from the unbiased simulation and (2) the position-dependent average force is then subtracted from the instantaneous force, adapting over sampling time. After sufficient sampling the energy surface along ξ is effectively flattened, removing energy barriers along the colvar, and the system will diffuse as a result of the random fluctuating force acting on it along the other degrees of freedom. Free energy changes are then calculated by integrating the average forces along ξ. Free energy calculations on the junction systems were divided into five windows, sampling from -5 Å to 10 Å RMSD (−90° to +90°) along ξ. The windows covered the intervals [−5, −1], [−2, −3], [1, 6], [4, 8] and [6, 10], and each window was subdivided into 15 bins for the force calculations; generating the PMF at 46 discrete points. Harmonic wall restraints were applied at the edges of the windows to keep the solute within the desired region. Initial ABF simulations of 100 ns per window were performed for each of the four junction systems, followed by additional runs in under-sampled bins to ensure a minimum per-bin sampling of 5 ns. Blocking analysis, using the method of Flyvbjerg and Petersen (101), was performed on projections of the unbiased MD 4WJ trajectories onto the second slowest essential mode (*J*_*roll*_) to obtain a decorrelation time of ∼4 ns, thus the minimum sampling was set to 5 ns (Supplementary Figures S44 and S45). The average sampling times per bin were 10.2 ns (J1^1^), 10.4 ns (J1^2^), 10.1 ns (J24^1^), and 10.6 ns (J24^2^). In addition, an identical blocking analysis was performed on the slowest essential mode (*J*_*twist*_), where a decorrelation time of ∼32 ns was found, confirming the choice of using 60 ns of each trajectory for system equilibration (Supplementary Figures S46–S47). An upper limit for the standard error in the ABF calculations, *SD*[Δ*A*^*ABF*^] (88), for a free energy difference, Δ*A*^*ABF*^, between points ξ_*a*_ and ξ_*b*_ along the colvar, ξ, was estimated as:

(13) $$\begin{equation*} SD[ \Delta A^{ABF} ] \approx (\xi _b - \xi _a ) \frac{\sigma }{K^{1/2}} (1 + 2\tau )^{1/2} \end{equation*}$$

where σ^2^ is the variance of the force along ξ, *K* is the total number of force evaluations and τ is the correlation length for the calculated force.

### Experimental determination of immobile junction isomer distributions

The distribution of crossover isomers in J1 has been reported previously (27), and the same method was used to determine the isomer distribution in J24. Briefly, strands 1 and 4 were labeled at their 5′ ends with fluorescein and tetramethylrhodamine, respectively. The distance between fluorescein (donor) and tetramethylrhodamine (acceptor) was probed by time-resolved Förster resonance energy transfer (tr-FRET). When the Iso I structure is adopted, the dyes are expected to be relatively close in space (Figure 1A), giving rise to a relatively fast rate of energy transfer. In contrast, if the Iso II structure is adopted, the dyes will be located at opposite ends of a duplex stacking domain (Figure 1A), resulting in a relatively long donor–acceptor (D–A) distance and a slower rate of energy transfer. Additionally, if both isomers are coexistent, then two sub-populations with distinct energy transfer rates will be present. To resolve this potential heterogeneity, the fluorescein donor was excited with a short laser pulse at 514 nm and the resulting decay of donor emission (at 540 nm) was recorded over time by time-correlated single photon counting. The donor intensity decay was fitted with an expression incorporating two Gaussian distributions of D-A distances, one for each crossover isomer, and was weighted according to the equilibrium fractions of each. The expression also included the intrinsic decay times of the donor, which were determined independently using a sample that lacked the tetramethylrhodamine acceptor. During the analysis, the equilibrium fraction of Iso I (*fr*_*IsoI*_) was optimized for best fit (note that *fr*_*IsoII*_ = 1 − *fr*_*IsoI*_). Additional details of this analysis are described in Miick *et al.* (27).

## RESULTS AND DISCUSSION

### Immobile 4WJs deviate from canonical B-form structure

The six base-pair step helical parameters (shift, slide, rise, tilt, roll and twist) provide a useful, widely-adopted coarse-grained description of DNA (58). As sequence has known effects on DNA structure, we sought to extend these six helical parameters to the 4WJ motif to highlight regions of interest. The deviations from reference canonical B-form duplex simulations of the junction pseudo-duplex arms were calculated for both isomers of J1 as well as for several topological variants of the J1 sequence, as detailed in Methods. A hexagonal plot representation (Figure 2A) is used to show the deviations in all six parameters for each given step in a particular junction/variant system. Two sets of scale bars are shown as rings within each plot, the inner representing a deviation of 0.5 Å for translational parameters (shift, slide, and rise) and a deviation of 5° for rotational parameters (tilt, roll, and twist); the outer representing deviations of 1.0 Å and 10°, respectively.

The J1 system exhibits pronounced negative mean parameter changes at the core step (step 5) and smaller deviations are present up to two neighbouring steps away (Figure 2B). Negative shift is observed in all core steps along with large untwisting (negative twist) of the pseudo-duplexes on one helix per isomer at step 5. Notably, the untwisting effect is localized on the CT base-step in isomer 1 and the TG base-step in isomer 2. Rupturing one crossover link removes most of these deviations with the SXB (Supplementary Figure S1) and SXD (Supplementary Figure S2) systems showing only slight relative changes compared to the full junction, and the nicked-duplex systems (Supplementary Figure S3) behave almost exactly like B-form DNA in terms of mean step parameter values. Line plots of all the absolute mean values for the J1 systems are included in the Supplementary information (Supplementary Figures S7–S10).

Similarly, the core steps of the J24 junction system (Figure 2C) show significant untwisting at step 5 (J24$_{(1,5)}^1$, J24$_{(1,5)}^2$ and J24$_{(2,5)}^2$). In isomer 1, the untwisting effect is localized on the CA base-step, and in isomer 2, the untwisting effect occurs on both the TA and GG base-steps. A major difference between the J1 and J24 systems is seen when rupturing one crossover link to form the SXB (Supplementary Figure S4), SXD (Supplementary Figure S5), and nicked-duplex (Supplementary Figure S6) systems. Unlike the J1 system, the J24 SXB variant maintains the large untwisting effect at step 5, and in isomer 2 it is now localized on the GG base-step only. Conversely, the SXD and nicked-duplex systems for J24 show only slight changes from B-form DNA. Line plots of all absolute mean values for J24 systems are included in the supporting information (Supplementary Figures S11–S14).

The results presented here indicate a local-sequence effect is present as deviations are occurring at base-pair steps of known high flexibility, i.e. untwisting of the pyrimidine-purine TG steps at J1$_{(1,6)}^1$ and J1$_{(2,5)}^2$ and the TA step at J24$_{(1,5)}^2$. Changes in mean values are also apparent at the comparatively stable CT and GG steps at J1$_{(1,5)}^1$ and J24$_{(2,5)}^2$ respectively, indicating a global topological effect. Step 5 in isomer 1, helix 1 and isomer 2, helix 2 in both junction systems is deformed, irrespective of relative flexibility, suggesting that the geometric constraints of the 4WJ motif are contributing as well as the local sequence effects. In both the J1 and J24 systems, these results seem to indicate that the formation of the 4WJ introduces tension that is relieved primarily in the shift and twist base-step parameters. It should be noted that as J24 has some local sequence differences to J1 around the core, reference B-form values were taken from literature simulations (56) for these steps. A comparison of the B-form simulation-obtained values in this work with these literature values (Supplementary Figures S15–S22) shows that the slight differences in solvent and temperature conditions between the two sets of CHARMM simulations has a negligible effect on the mean values and hence the comparisons are reasonable.

### Flexibility of base-pair step parameters

In addition to comparing the relative values of base-pair step parameters for topological variants of the J1 and J24 4WJ motif, it is useful to compare the deviations in stiffness constants between topological variants for the base-pair step parameters. The effect of junction topology upon base-pair step parameter flexibility is shown in Figure 3 for the J1 and J24 variants. Unsurprisingly, in all variants, the stiffness constants are less than those in B-form DNA, meaning they exhibit greater flexibility due to the presence of the junction. In addition, where base-pair step absolute deviations (Figure 2) are primarily found on step 5, deviations in flexibility are exhibited throughout the junction (although mostly on steps 4 through 6). The major untwisting effect is shown as greater flexibility of twisting in all base-pair steps in the junctions.

The flexibility of the topological variants offer some insight into the deviations between the J1 and J24 systems. Both single-crossover variants of J1 show minimal deviations in twist flexibility from B-form DNA, but the SXB variant of J24 exhibits a similar increase in twist flexibility as the 4WJ, which is not shown in the SXD variant. These observations match with those seen when comparing absolute base-pair step deviations, as the SXD variant exhibits greater stability than SXD in the J24 system. Interestingly, in both J1 and J24, the nicked variants show an increase in twist flexibility not necessarily seen in the single-crossover variants, showing that one crossover in some cases is more stable than the four-way junction. Line plots of the stiffness constants for the J1 and J24 variants are found in the supporting information (Supplementary Figures S23–S30), as well as a comparison of the B-form (duplex) simulation-obtained values in this work with literature values (Supplementary Figures S31–S38).

### Base-pair step deformability is a consequence of both topology and local sequence

**Figure 4.**
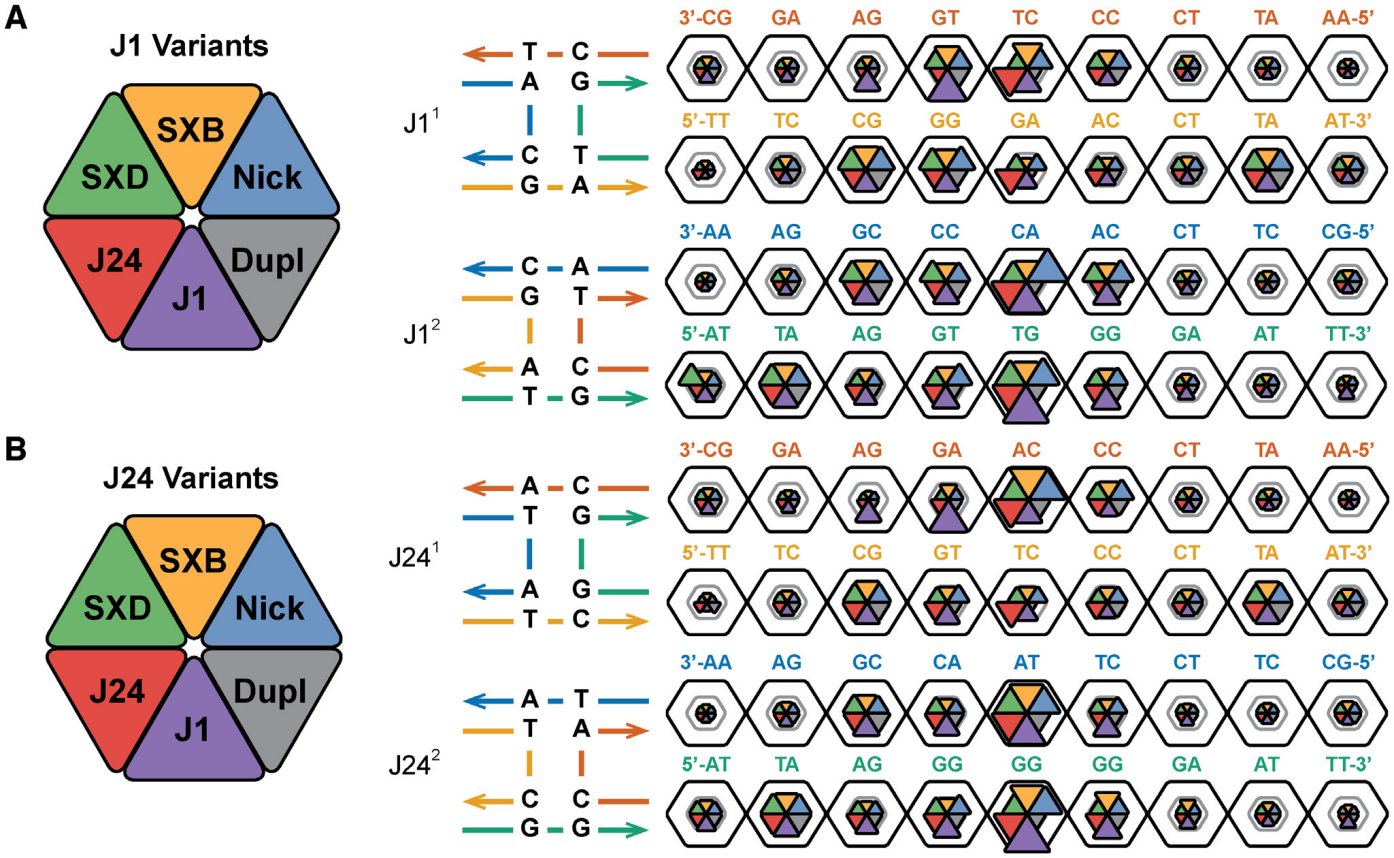
Junction base sequences confer unique base-pair step deformability. (**A**, **B**) The configurational volumes for all six topological variants of J1 and J24 are shown as wedges in a hexagonal plot (left). The inner gray line in each hexagon represents a volume of 10 Å^3^·deg^3^; the outer black line represents deviations of 100 Å^3^·deg^3^. Topologies of the J1 and J24 junction systems are shown to the left and right of the dotted line. Isomer 1 is shown on the top and isomer 2 on the bottom for both core sequences. Base-pair step sequences are shown for strands A (red in isomer 1, blue in isomer 2) and C (orange in isomer 1, green in isomer 2); the 5 to 3 sequence runs right to left for helix 1 and left to right for helix 2 for both isomers.

In order to obtain a full description of base-pair step deformability, the off-diagonal coupling terms of the stiffness matrix need to be considered (51). The configurational volume metric, calculated as the product of the eigenvalues of the covariance matric in helical parameter space, has been used to characterize both X-ray crystallography and MD data (50,51) and is used here to summarize the relative deformabilities of each of the analyzed systems. A modification of the hexagonal plots in Figures 2 and 3 is used to compare the per-step configurational volumes of the different topological (duplex, nicked-duplex, and single-crossovers) and sequence variants (J1, J24) in Figure 4. The two log_10_ scale bar rings represent volumes of 10 and 100 Å^3^·deg^3^, respectively.

The sequence-level heterogeneity in step flexibility is immediately apparent, i.e. the flexible TA step at step 8 in isomer 1, helix 2 compared to the rigid AT step at step 8 in isomer 2, helix 2 in all systems. The effect of changes in local sequence can be seen in how the TG step at J1$_{(1,6)}^1$ is significantly more flexible in this location than when moved to J1$_{(2,4)}^2$ where both have the same proximity to the junction. The increased flexibility at J1$_{(1,6)}^1$ compared to other topological variants also indicates the significance of topology on local DNA structure. The J1$_{(1-2,5)}^1$ steps have notably lower flexibilities, by approximately an order of magnitude, than their J1$_{(1-2,5)}^2$ equivalents with the same relative relationship present between their N1$_{(1-2,5)}^1$ and N1$_{(1-2,5)}^2$ counterparts, though at slightly lower volume values. As the J1^1^ isomer is known to be significantly favoured over J1^2^, this result is significant as it suggests a relative energetic stability at the core in J1^1^ and this will be further investigated in the following section. Also, as the nicked-duplex systems show a similar pattern to the J1 isomers, this effect is likely due to both the local sequence at the junction core and the geometric constraints applied by the 4WJ motif. The SX systems exhibit generally smaller deformabilities at the core than their junction and nicked-duplex equivalents, except at step 5 in isomer 2, helix 2 where they are as flexible as their counterparts. The two systems do show distinct deformability distributions, however, in agreement with their mean value deviations and this suggests that their topology has an effect on local structure. The relative stability of single-crossovers, at least in isolation, is in agreement with their extensive and successful use in tile-based applications (8).

The J24 sequence shows increased deformability in both isomers at the junction core, with less propagation to neighbouring steps, in contrast to J1 which exhibits increased deformability up to two neighbours from the core. In addition, the deformability at the core of both J24 isomers is similar, unlike in J1 where isomer 2 is more flexible than isomer 1. This result suggests a similar energetic stability of both J24 isomers. The effect of local sequence can also be seen in the major reduction in configurational volume when the TG step J1$_{(1,6)}^1$ is changed to an AG step in J24$_{(1,6)}^1$. In most cases, the TA and CG base-pair steps show the greatest flexibility even far from the junction core. Line plots of the configurational volume values are included in Supplementary Figures S39 and S40.

### Free energies of isomerization

The significant differences in configurational volumes between the isomers of J1 and the comparable values for those of J24 suggest that unique atomic-level interactions are occurring at or near the 4WJ cores. To unpack the various contributions to the junction energy of isomerization, ΔΔ*E*_*iso*_ = Δ*E*_*Iso*1_ − Δ*E*_*Iso*2_, the MM-PBSA end-state free energy analysis method was applied to structural ensembles of each isomer. Supplementary Figure S41 shows plots of the various per-base energy contributions to the total isomerization energy for each chain in J1 and J24, respectively. J1^1^ is more stable than J1^2^ in all energy terms (Δ*E*_*INT*_, Δ*E*_*VDW*_, Δ*E*_*NP*_, Δ*E*_*P*_) except gas-phase electrostatics (Δ*E*_*ELE*_), though this is offset by the large polar-solvation energy difference to give a total energy difference of -8.2 kcal/mol. It should be noted that all energy totals presented here do not include the solute entropy contribution as the quasiharmonic approximation has not converged (Supplementary Figure S42), though these contributions would most likely favour the more deformable systems in Figure 4A and B. The plots of Δ*E*_*TOT*_ in Supplementary Figure S41B show that strands I, III, and IV are all more stable in isomer 1 at the core bases, with only strand II favouring isomer 2 at bases 7 and 8. These two bases, along with the non-core base 6, correspond to the region of high deformability at J1$_{(1,5-7)}^1$ around the flexible TG step in Figure 4A. The largest energy differences occur at the core bases (indices 7-10) where bases are being changed from a linear configuration (chains I + III in isomer 1, II + IV in isomer 2) into a ‘bent’ configuration (II + IV in isomer 1, I + III in isomer 2) though energy changes are also seen at neighbouring bases. In the J24 system the isomers are energetically similar, with J24^1^ being favoured in the Δ*E*_*VDW*_, Δ*E*_*NP*_ and Δ*E*_*P*_ terms and J24^2^ in Δ*E*_*INT*_ and Δ*E*_*ELE*_ for a total energy difference of 0.4 kcal/mol. Looking at the Δ*E*_*TOT*_ plots for J24, strands I + III favour isomer 1 at the core, while II + IV favour isomer 2. The J24 energy changes are more focused at the core than J1, consistent with the reduced deformability of non-core bases in Figure 4A relative to their J1 counterparts. The data confirms that stacking energies alone, represented by the Δ*E*_*VDW*_ term, are not the only interactions that change upon isomerization as both geometric (Δ*E*_*INT*_) and electrostatic (Δ*E*_*ELE*_ + Δ*E*_*P*_) factors contribute to the total energy difference. Furthermore, the presence of electrostatic interactions is in agreement with the previously suggested hypothesis of a sequence-dependent electrostatic potential contributing to the isomerization energy (37,49) and is consistent with the results of Mg^2 +^ pulsing experiments (45).

### Experimental conformer ratios of J1 and J24 immobile junctions

The free energies of isomerization from molecular dynamics ensemble measurements can be directly compared to the conformer ratios of J1 and J24 from tr-FRET and NMR experiments. The distribution of crossover isomers in J1 has been previously reported where the fraction of isomer 1 was ≥0.95 ± 0.05 and the fraction of isomer 2 was ≤0.05 ± 0.05, for an isomer ratio of ≥19:1 (I/II:I/IV) (27). The conformer ratios and free energies of isomerization of the J1 and J24 immobile junctions are compared in Table 1 below. Here the resulting isomer ratios for the J24 junction are a fraction of isomer 1 of 0.49 ± 0.05 and a fraction of isomer 2 of 0.51 ± 0.05, for an isomer ratio of 1:1 (I/II:I/IV). The free energy differences between isomers 1 and 2 can be determined according to their isomer ratios by using Equation (14), resulting in a value of ≤–7.2 kJ/mol ( ≤–1.71 kcal/mol) for J1 and 0.10 kJ/mol (0.02 kcal/mol) for J24.

(14) $$\begin{equation*} \Delta \Delta G = -R T \rm {ln}(fr_{IsoI}/fr_{IsoII}) \end{equation*}$$

In the previous section, the free energies of isomerization of the J1 and J24 junctions were calculated using the MM-PBSA free energy analysis method on the structural configurations from MD simulations. From these simulations, the free energies of isomerization were ΔΔ*E*_*iso*_ = −8.2 kcal/mol for J1 and ΔΔ*E*_*iso*_ = 0.4 kcal/mol for J24, comparable to the trend seen in the experimental ratios where isomer 1 is strongly favored in J1 and both isomers are favored equally in J24. The differences in the absolute values of free energy of isomerization from simulation to experiment can be potentially attributed to the solute entropic energies from the simulation remaining unconverged after 300 ns (Supplementary Figure S42), leading to higher absolute magnitudes of the free energies of isomerization.

**Table 1.** Conformer ratios of the J1 and J24 immobile Holliday junctions

	Stacking partner		Mean ratio (%)^a^		ΔΔ*G°*^, b^
	I/II+IV/III	II/III+I/IV	I/II	I/IV	(kcal/mol)
J1^c^	A/G+T/C	G/T+C/A	≥95 ± 5	≤5 ± 5	≤−1.71
J24	T/G+G/A	T/A+C/C	49 ± 5	51 ± 5	0.02

^a^These columns show the conformer ratios ($\%$) of the Holliday junctions. Each value is an average of several independent tr-FRET measurements. ^b^The free energy differences of stacked conformer I/II to I/IV were calculated from their conformer ratios according to Equation (14). Negative values indicate that the I/II+IV/III conformer is thermodynamically favored. ^c^Data previously determined both from tr-FRET and NMR (27).

### Core sequences exhibit unique junction dynamics

**Figure 5.**
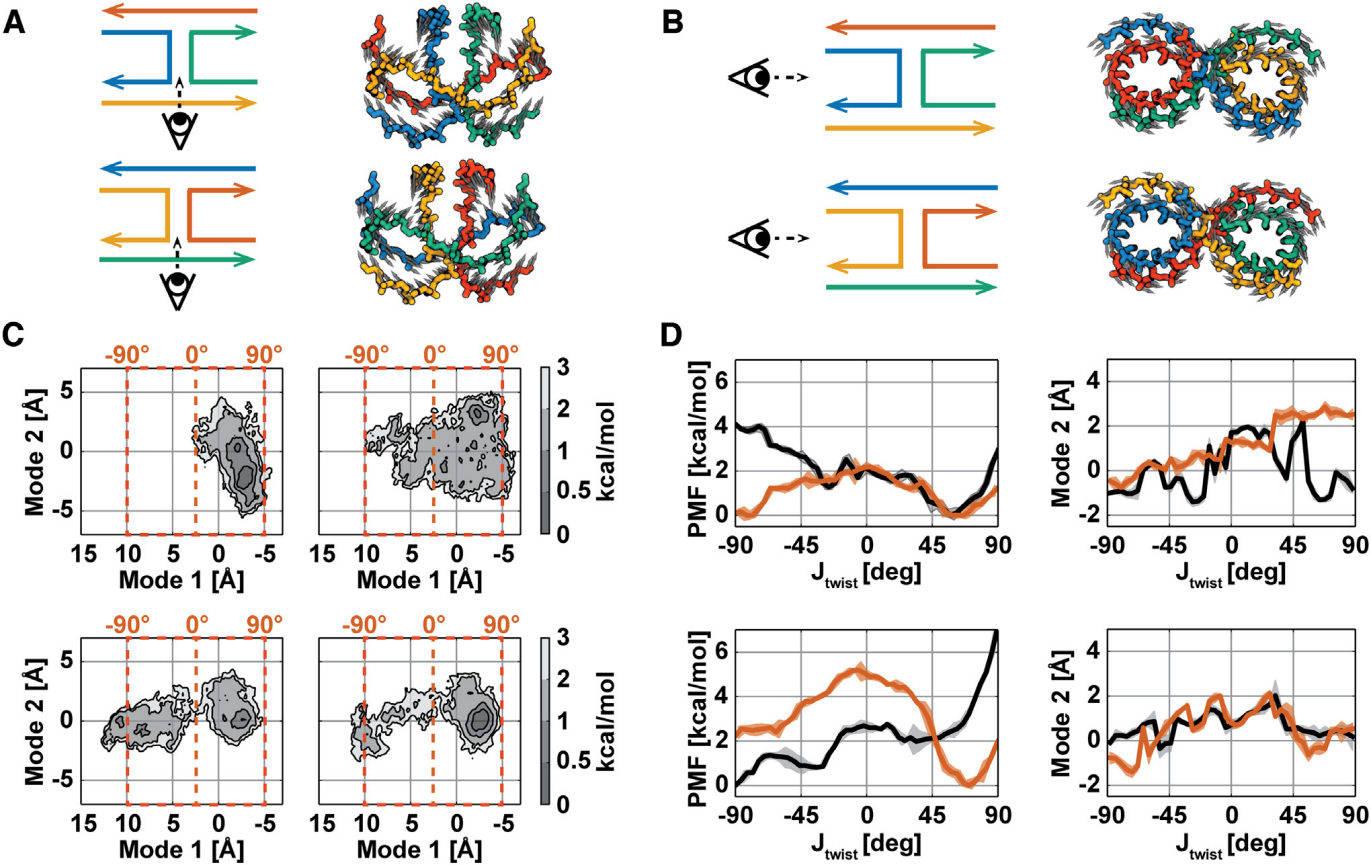
Junction stacking interactions confer unique global system dynamics. (**A**) The eigenvector components of the highest variance mode (Mode 1) are shown as gray arrows on a rendering of the average meta-ensemble backbone structure, coloured for isomer 1 (top) and isomer 2 (bottom). (**B**) The eigenvector components of the second highest variance mode (Mode 2) are shown as gray arrows on a rendering of the average meta-ensemble backbone structure, coloured for isomer 1 (top) and isomer 2 (bottom). (**C**) Projections of the unbiased simulations onto these eigenvectors are shown for J1 (top row) and J24 (bottom row). For each system projection isomer 1 is shown on the left and isomer 2 is shown on the right. Contours are shaded according to free energy values calculated relative to the most populated bin for each system; bin areas are 0.04 Å^2^ each. Corresponding *J*_*twist*_ values are shown in orange for Mode 1. **(D)** Potentials of mean force, from independent ABF simulations to (C), calculated along Mode 1 for isomer 1 (black) and isomer 2 (orange) are shown for J1 (top, left) and for J24 (bottom, left); the shaded regions represent the error calculated with Equation (13). The average value of Mode 2 in each sampling bin for isomer 1 (black) and isomer 2 (orange) are shown for J1 (top, right) and for J24 (bottom, right); the shaded regions represent the per-bin standard deviations.

As DNA nanostructures are often comprised of arrays of interconnected 4WJs, knowledge of sequence effects on global dynamics is necessary to exert a fine control over a nanodevice. PCA was performed on the Cartesian coordinates of all the equilibrated junction data, 2.9 μs in total, to obtain the essential dynamical modes of an averaged 4WJ. The highest variance modes were found to be the in-plane scissor-like motion, *J*_*twist*_ (Figure 5A), and the rolling of the junction arms, *J*_*roll*_ (Figure 5B). Definitions of these motions have both been reported in crystallographic studies (32,74) and their contributions to the total variance are shown in Supplementary Figure S43. While J1^2^, J24^1^ and J24^2^ all show predominant motion along *J*_*twist*_, J1^1^ shows predominant motion along *J*_*roll*_. In addition, projections of the unbiased trajectories onto the two principal modes reveal all junctions except J1^1^ show movement to left-handed configurations (negative twist) during the various simulation replicates (Figure 5C). We speculate that the large-scale motions involved in the *J*_*twist*_ mode are responsible for configurational switching from isomer 1 to isomer 2 and vice versa. As J1^1^ is the most stable isomer in this study, it is perhaps not surprising that *J*_*twist*_ is less predominant for this isomer only. This sequence-dependent configurational heterogeneity is consistent with previous NMR studies performed on various permutations of junction core sequence (27). Enhanced sampling along the *J*_*twist*_ eigenvector, obtained from PCA, was performed using the ABF algorithm to determine the PMFs along this reduced coordinate in the antiparallel regime [-90°, +90°] (37) in a new set of biased simulations. A minimum sampling of 5 ns was performed in each bin as this ensures that the unbiased degrees of freedom have de-correlated, where the slowest relaxation time, that of *J*_*roll*_, is ∼4 ns (Supplementary Figures S44 and S45). The PMFs show that each junction has a unique flexibility and handedness along *J*_*twist*_ where J1^1^ prefers to be right-handed, and J1^2^ shows equal preference for right- and left-handedness (Figure 5D). The J1 isomers have very different average rolls along the *J*_*twist*_ coordinate, while the J24 isomers have fairly similar roll profiles. This result is significant as even in the presence of equal energy isomers (J24), there can be unique sequence-dependent global dynamics.

## CONCLUSIONS

Analyses of explicit solvent MD simulations of a reference J1 and J24 junction sequence, as well as duplex, nicked-duplex, single-crossover, and junction-core sequence analogues, were used to demonstrate that asymmetric immobile 4WJs deviate from canonical B-form DNA, both in structure and, to a larger degree, flexibility. In particular, the sequence differences between the J1 and J24 manifest as distinct isomerization preferences. The chemical topology and local base-pair sequence were shown to contribute to these structural changes both in the 4WJ motif and in its topological variants. Free energy analyses showed that stacking, geometric, and electrostatic factors all contribute to the energy difference between stacked junction isomers and that these contributions can come from both core and non-core bases. The distinct free energies of isomerization for J1 and J24 are confirmed using isomeric ratio analysis from tr-FRET measurements. Finally, enhanced sampling MD revealed that junction core base stacking sequences exhibit unique and significant global junction dynamics that are independent of isomerization energy differences.

Tentative sequence design guidance for larger DNA assemblies can be garnered from the results contained here. First, the natural deformabilities of the various B-form base-pair steps are exacerbated at or near crossover structural motifs, making this relevant in DNA nanotechnology design. If rigidity of a nanostructure is desired, the flanking sequences of these motifs should be selected from the more rigid purine–pyrimidine tetranucleotide sequences. On the other hand, should flexibility be desired, the pyrimidine–purine tetranucleotide sequences might be preferable. At the core, certain base-pair steps appear to be more deformed as a consequence of geometry, irrespective of sequence, and so energetically it may be best to select bases with flexibilities along specific degrees of freedom (e.g., twist and slide) for these locations, with rigid bases at the other core locations. In future work, these hypotheses could be tested, ideally with free energy perturbation (FEP) methodologies.

In this study, the J1 and J24 junctions were chosen due to their isomerization tendencies, where J1 has an energetic preference towards isomer 1 and J24 has a roughly equal energetic preference for both isomers. At this time, no detailed structural information is available from experiment for the J1 or J24 junctions, so a base-pair level comparison of structure and dynamics is not possible. Future work may investigate the sequence effect on isomerization of a broader range of junctions, including those with detailed structural data from experiment (44). Knowledge of the energetic preference of isomerization of different junctions could play an important role in the design of nanostructures, particularly when only several junctions are present, compared with the many dozens present in scaffolded DNA origami. In effect, the sequence of the junction could be designed for the specific role of the junction in the overall assembly. Future work may additionally investigate any cumulative effects of sequential and parallel junction topologies on larger-scale nanostructures. In particular, future large-scale MD studies, including coarse-grained MD, will likely benefit from accurate conformational and energetic data at the junction-level.

## DATA AVAILABILITY

All data is available upon request from the corresponding authors.

## Supplementary Material

gkab1246_Supplemental_File

## Notes

Present address: Guo Qing Tang, Ra Pharmaceuticals, Cambridge, MA 02140, USA.
