# Supplementary material for: Computational investigation of the impact of core sequence on immobile DNA four-way junction structure and dynamics
Source: Nucleic Acids Res. 2021 Dec 22;50(2):717–30. doi: 10.1093/nar/gkab1246 (PMC8789063; doi:10.1093/nar/gkab1246)
Supplement: gkab1246_Supplemental_File [file gkab1246_supplemental_file.pdf]

Supporting Material: Computational investigation of the impact of core sequence on immobile DNA four-way junction structure and dynamics

Matthew R. Adendorff,[†] Guo Qing Tang,[‡,¶] David P. Millar,[§] Mark Bathe,[*,†] and William P. Bricker[*,†,‖]

†*Department of Biological Engineering, Massachusetts Institute of Technology, Cambridge, MA 02139, USA*

‡*Department of Molecular Biology, Scripps Research Institute, La Jolla, CA 92037, USA*

¶*Current affiliation: Ra Pharmaceuticals, Cambridge, MA 02140, USA*

§*Department of Integrative Structural and Computational Biology, Scripps Research Institute, La Jolla, CA 92037, USA*

‖*Department of Chemical and Biological Engineering, University of New Mexico, Albuquerque, NM 87131, USA*

E-mail: mark.bathe@mit.edu; wbricker@unm.edu

Phone: +1 (617) 324-5685; +1 (505) 277-6392

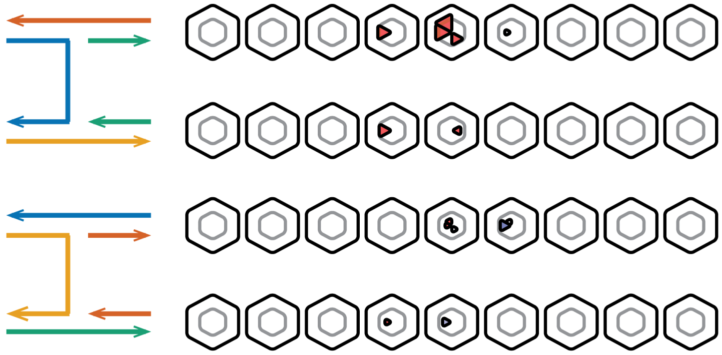

Figure S1: Deviations in step parameter mean values for the SXB junction topological variants of J1 (left) and their topologies (right). Deviations in mean parameter values from reference B-form simulations for each step are shown as wedges in a hexagon. The inner gray line represents a deviation of 0.5 Å for translational parameters (shift, slide, and rise) and a deviation of 5° for rotational parameters (tilt, roll, and twist); the outer black line represents deviations of 1.0 Å and 10° respectively. Negative deviations are coloured red, positive deviations are coloured blue, and the absence of a wedge indicates a deviation of less than 0.2 Å or 2°.

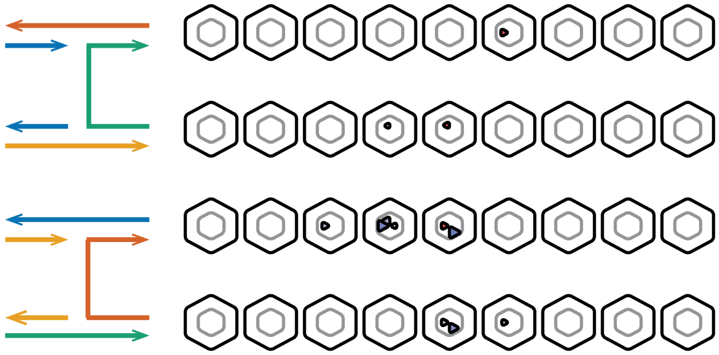

Figure S2: Deviations in step parameter mean values for the SXD junction topological variants of J1 (left) and their topologies (right). Deviations in mean parameter values from reference B-form simulations for each step are shown as wedges in a hexagon. The inner gray line represents a deviation of 0.5 Å for translational parameters (shift, slide, and rise) and a deviation of 5° for rotational parameters (tilt, roll, and twist); the outer black line represents deviations of 1.0 Å and 10° respectively. Negative deviations are coloured red, positive deviations are coloured blue, and the absence of a wedge indicates a deviation of less than 0.2 Å or 2°.

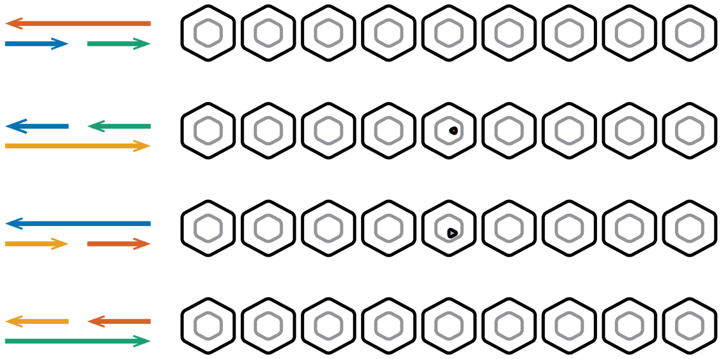

Figure S3: Deviations in step parameter mean values for the nicked duplex topological variants of J1 (left) and their topologies (right). Deviations in mean parameter values from reference B-form simulations for each step are shown as wedges in a hexagon. The inner gray line represents a deviation of 0.5 Å for translational parameters (shift, slide, and rise) and a deviation of 5° for rotational parameters (tilt, roll, and twist); the outer black line represents deviations of 1.0 Å and 10° respectively. Negative deviations are coloured red, positive deviations are coloured blue, and the absence of a wedge indicates a deviation of less than 0.2 Å or 2°.

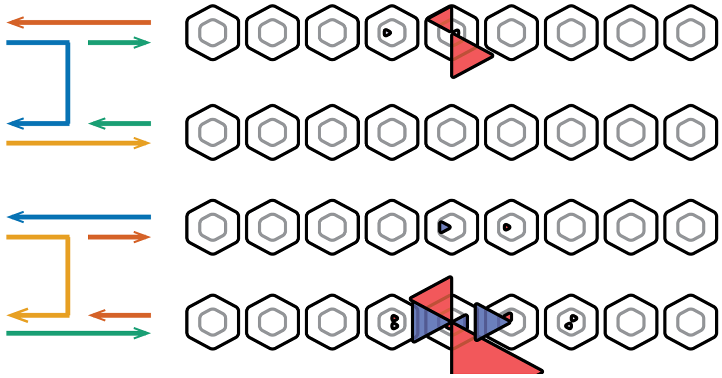

Figure S4: Deviations in step parameter mean values for the SXB junction topological variants of J24 (left) and their topologies (right). Deviations in mean parameter values from reference B-form simulations for each step are shown as wedges in a hexagon. The inner gray line represents a deviation of 0.5 Å for translational parameters (shift, slide, and rise) and a deviation of 5° for rotational parameters (tilt, roll, and twist); the outer black line represents deviations of 1.0 Å and 10° respectively. Negative deviations are coloured red, positive deviations are coloured blue, and the absence of a wedge indicates a deviation of less than 0.2 Å or 2°.

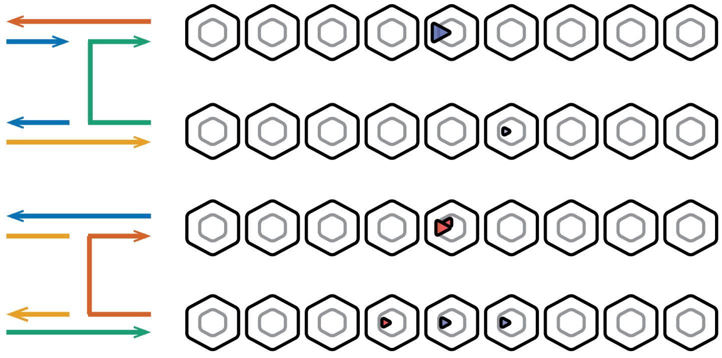

Figure S5: Deviations in step parameter mean values for the SXD junction topological variants of J24 (left) and their topologies (right). Deviations in mean parameter values from reference B-form simulations for each step are shown as wedges in a hexagon. The inner gray line represents a deviation of 0.5 Å for translational parameters (shift, slide, and rise) and a deviation of 5° for rotational parameters (tilt, roll, and twist); the outer black line represents deviations of 1.0 Å and 10° respectively. Negative deviations are coloured red, positive deviations are coloured blue, and the absence of a wedge indicates a deviation of less than 0.2 Å or 2°.

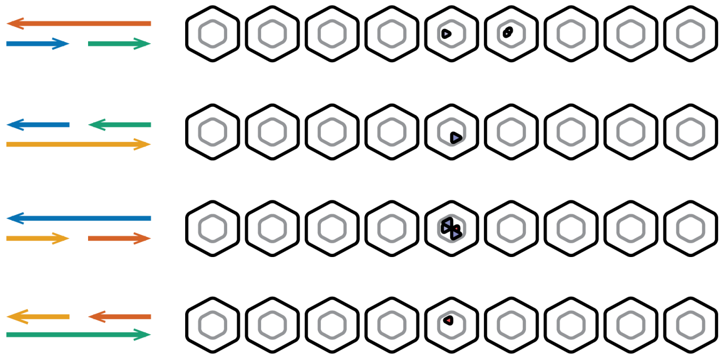

Figure S6: Deviations in step parameter mean values for the nicked duplex topological variants of J24 (left) and their topologies (right). Deviations in mean parameter values from reference B-form simulations for each step are shown as wedges in a hexagon. The inner gray line represents a deviation of 0.5 Å for translational parameters (shift, slide, and rise) and a deviation of 5° for rotational parameters (tilt, roll, and twist); the outer black line represents deviations of 1.0 Å and 10° respectively. Negative deviations are coloured red, positive deviations are coloured blue, and the absence of a wedge indicates a deviation of less than 0.2 Å or 2°.

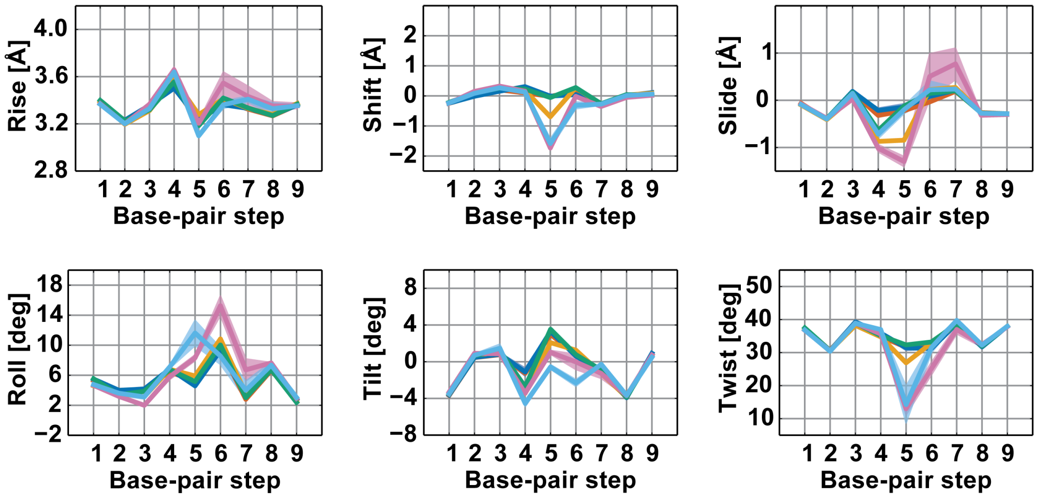

Figure S7: Mean values of the six base-step degrees of freedom for the inner nine base steps of helix 1 are shown for each of the different topologies of J1, as well as for the 4WJ topology of J24, in their isomer 1 forms. The various topologies are color coded as: red (duplex), dark blue (nicked-duplex), orange (SXB), green (SXD), red-purple (J1 4WJ), and light blue (J24 4WJ).

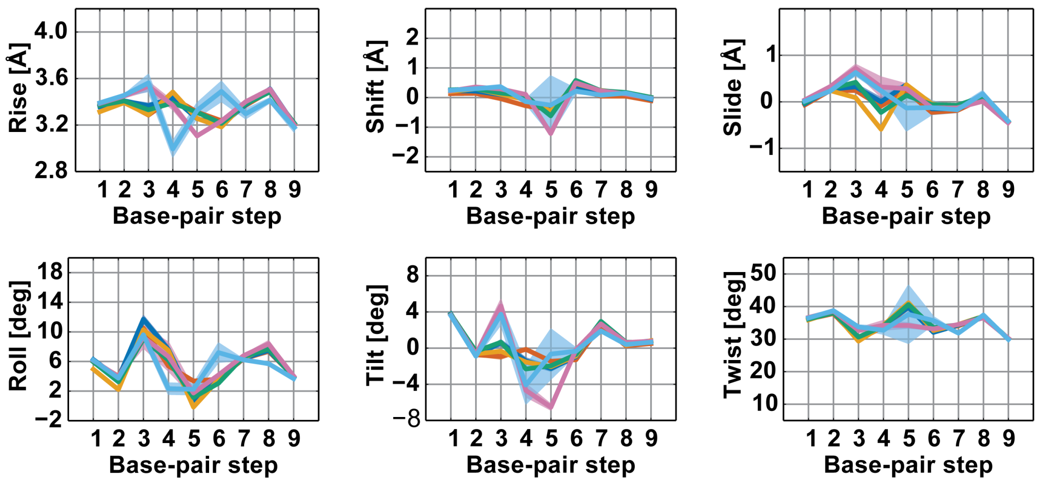

Figure S8: Mean values of the six base-step degrees of freedom for the inner nine base steps of helix 2 are shown for each of the different topologies of J1, as well as for the 4WJ topology of J24, in their isomer 1 forms. The various topologies are color coded as: red (duplex), dark blue (nicked-duplex), orange (SXB), green (SXD), red-purple (J1 4WJ), and light blue (J24 4WJ).

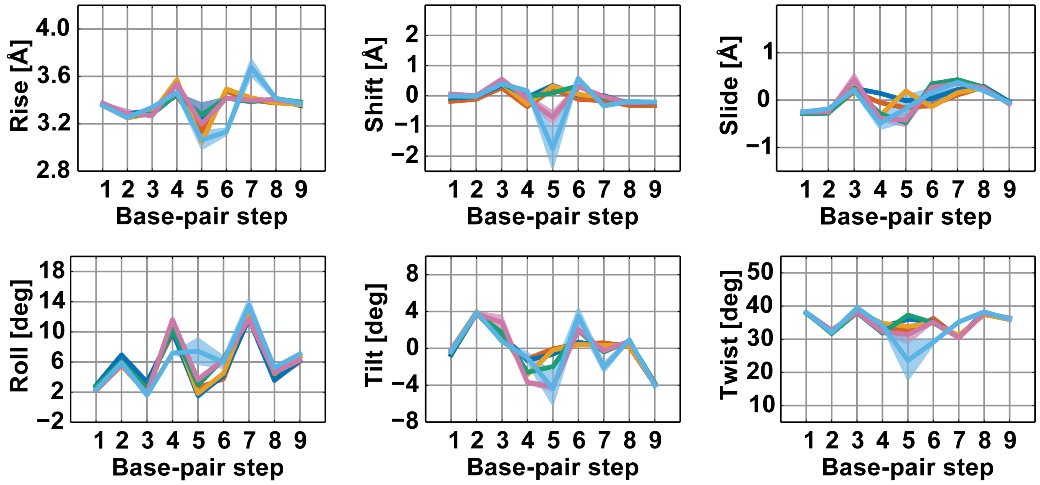

Figure S9: Mean values of the six base-step degrees of freedom for the inner nine base steps of helix 1 are shown for each of the different topologies of J1, as well as for the 4WJ topology of J24, in their isomer 2 forms. The various topologies are color coded as: red (duplex), dark blue (nicked-duplex), orange (SXB), green (SXD), red-purple (J1 4WJ), and light blue (J24 4WJ).

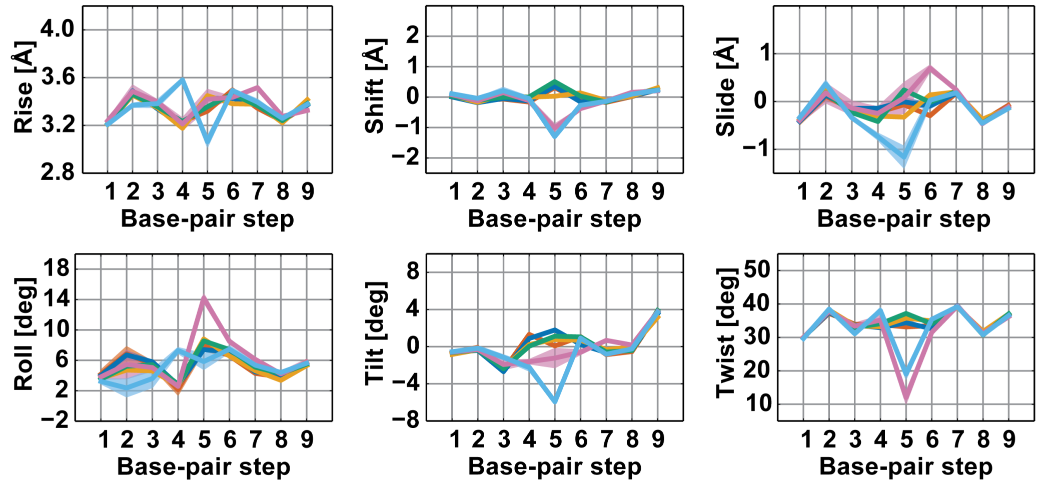

Figure S10: Mean values of the six base-step degrees of freedom for the inner nine base steps of helix 2 are shown for each of the different topologies of J1, as well as for the 4WJ topology of J24, in their isomer 2 forms. The various topologies are color coded as: red (duplex), dark blue (nicked-duplex), orange (SXB), green (SXD), red-purple (J1 4WJ), and light blue (J24 4WJ).

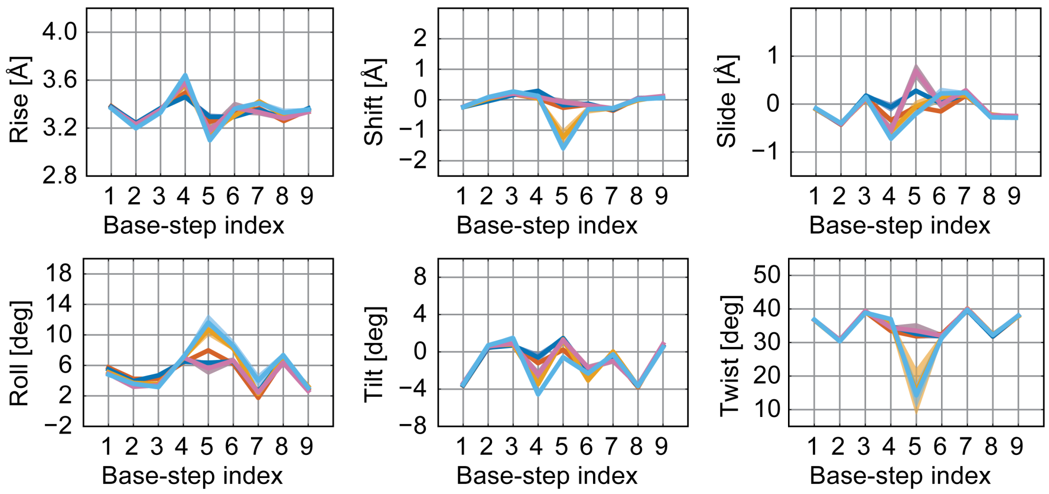

Figure S11: Mean values of the six base-step degrees of freedom for the inner nine base steps of helix 1 are shown for each of the different topologies of J24, as well as for the 4WJ topology of J1, in their isomer 1 forms. The various topologies are color coded as: red (duplex), dark blue (nicked-duplex), orange (SXB), green (SXD), red-purple (J1 4WJ), and light blue (J24 4WJ).

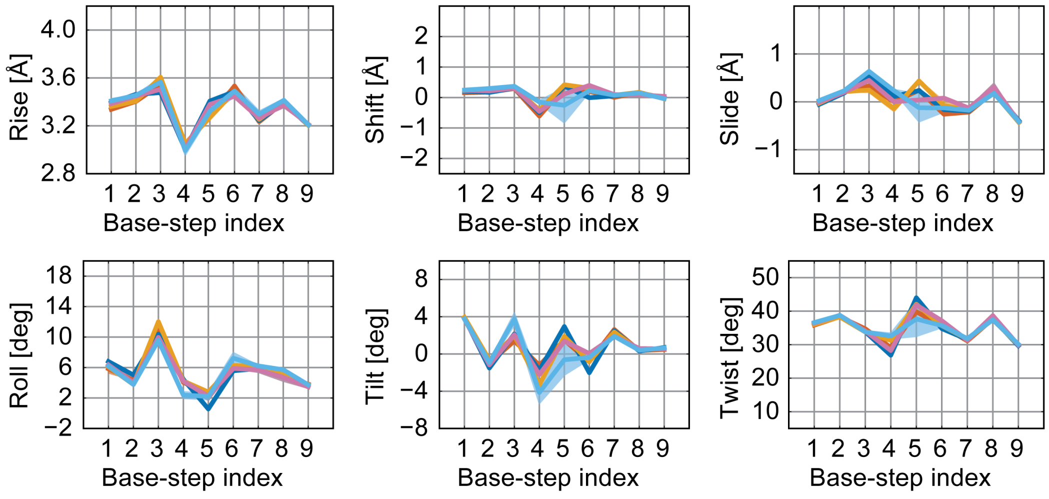

Figure S12: Mean values of the six base-step degrees of freedom for the inner nine base steps of helix 2 are shown for each of the different topologies of J24, as well as for the 4WJ topology of J1, in their isomer 1 forms. The various topologies are color coded as: red (duplex), dark blue (nicked-duplex), orange (SXB), green (SXD), red-purple (J1 4WJ), and light blue (J24 4WJ).

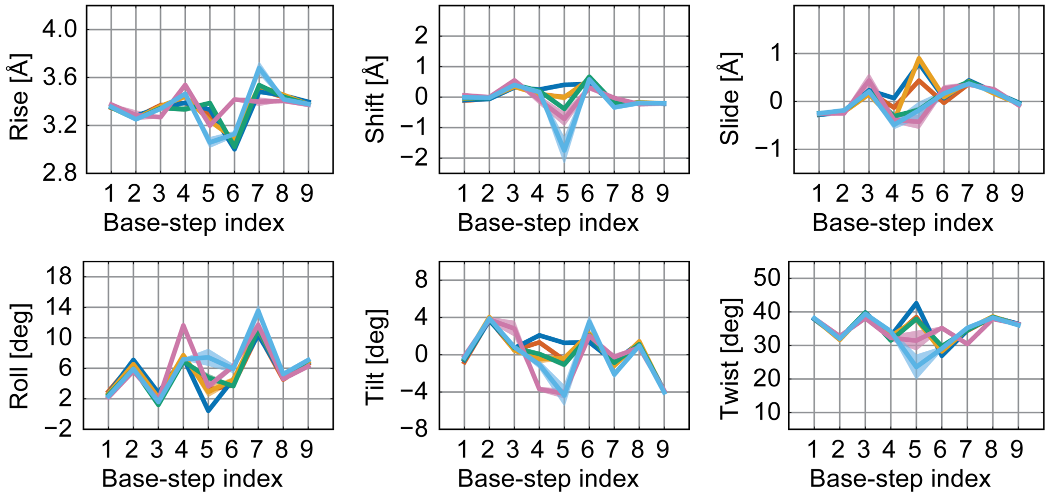

Figure S13: Mean values of the six base-step degrees of freedom for the inner nine base steps of helix 1 are shown for each of the different topologies of J24, as well as for the 4WJ topology of J1, in their isomer 2 forms. The various topologies are color coded as: red (duplex), dark blue (nicked-duplex), orange (SXB), green (SXD), red-purple (J1 4WJ), and light blue (J24 4WJ).

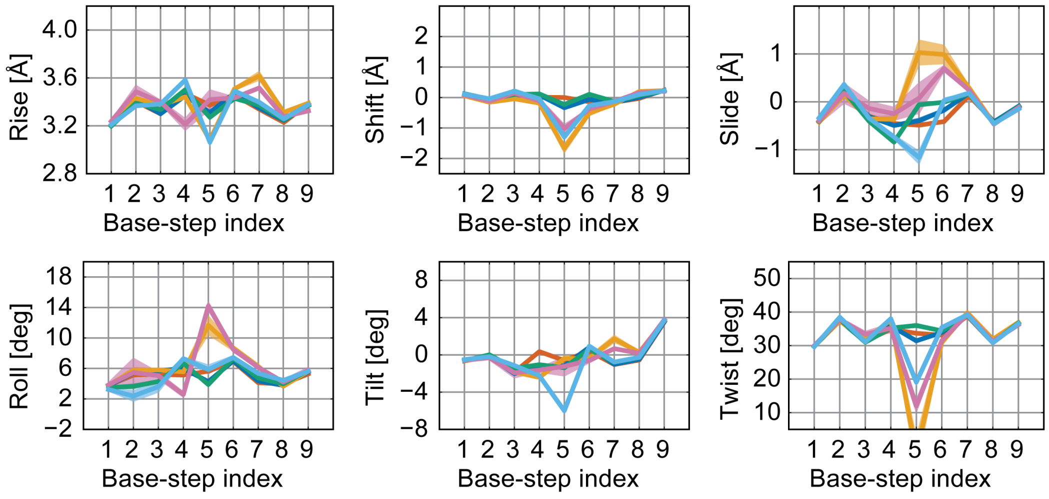

Figure S14: Mean values of the six base-step degrees of freedom for the inner nine base steps of helix 2 are shown for each of the different topologies of J24, as well as for the 4WJ topology of J1, in their isomer 2 forms. The various topologies are color coded as: red (duplex), dark blue (nicked-duplex), orange (SXB), green (SXD), red-purple (J1 4WJ), and light blue (J24 4WJ).

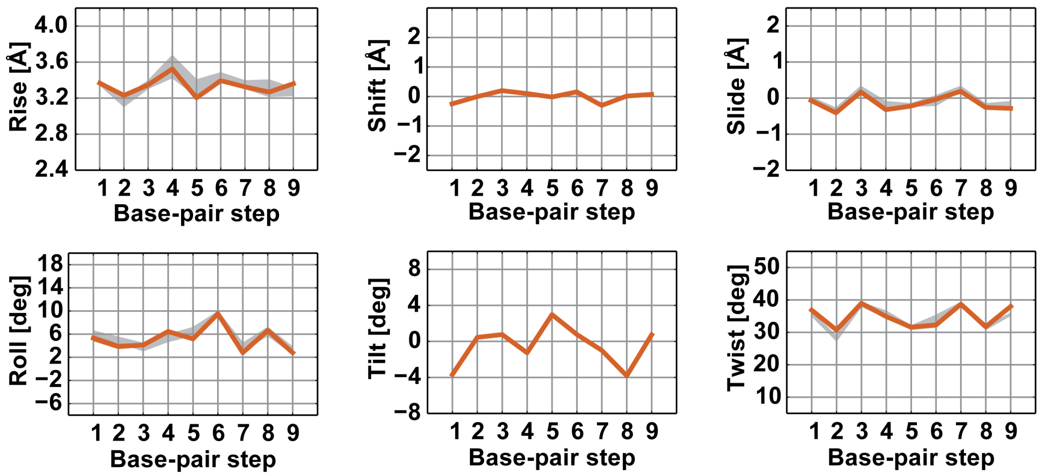

Figure S15: Comparison of the $J1^{1}_{(1)}$ duplex base-pair step parameter mean values with literature. The mean values from the present works simulations (red) are plotted with those from Dans et al, 2012 (gray). Shaded areas represent standard deviations. An absence of the reference line indicates complete overlap.

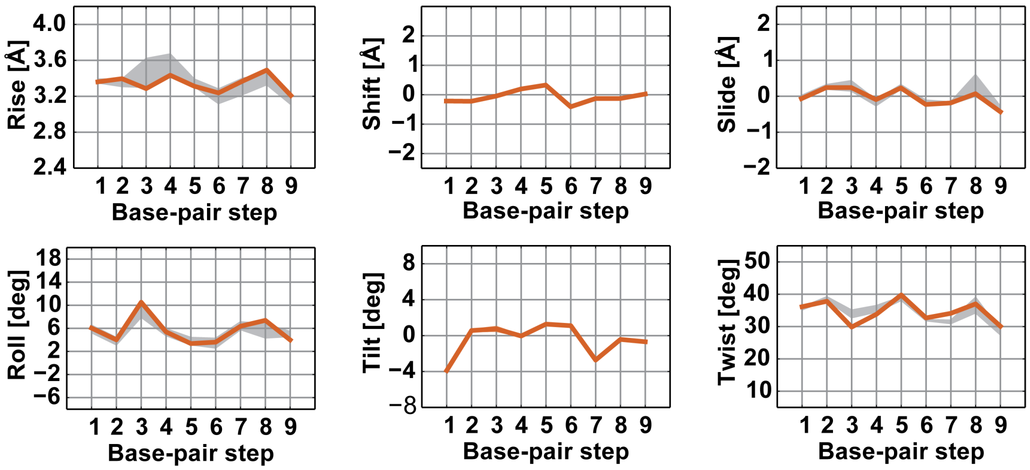

Figure S16: Comparison of the $J1^{1}_{(2)}$ duplex base-pair step parameter mean values with literature. The mean values from the present works simulations (red) are plotted with those from Dans et al, 2012 (gray). Shaded areas represent standard deviations. An absence of the reference line indicates complete overlap.

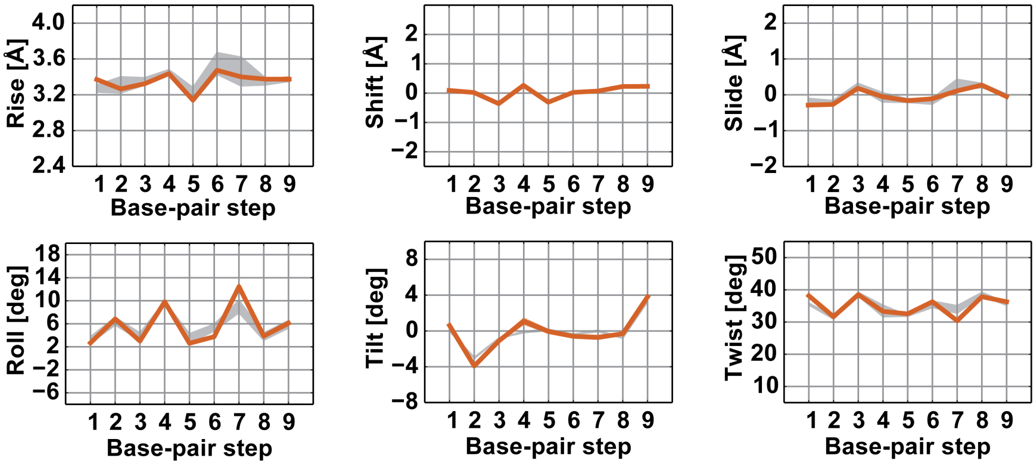

Figure S17: Comparison of the J1$^{2}_{(1)}$ duplex base-pair step parameter mean values with literature. The mean values from the present works simulations (red) are plotted with those from Dans et al, 2012 (gray). Shaded areas represent standard deviations. An absence of the reference line indicates complete overlap.

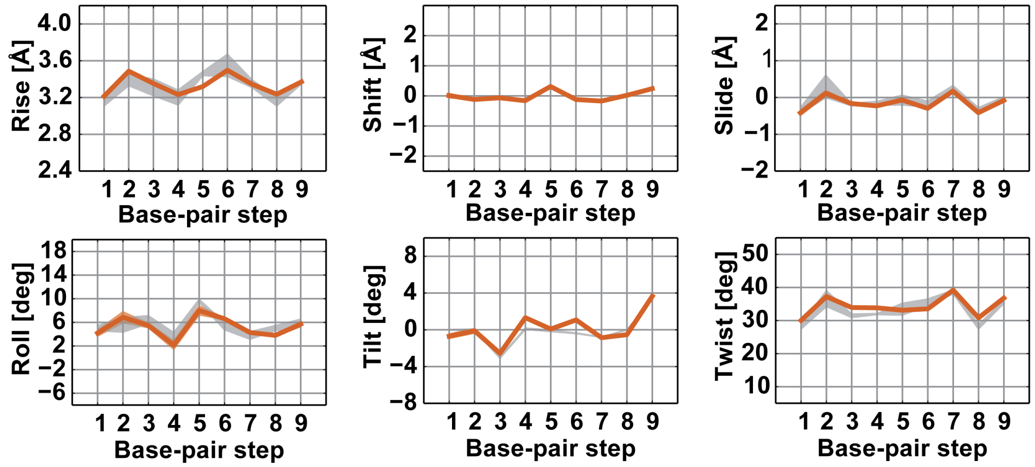

Figure S18: Comparison of the J1$^{2}_{(2)}$ duplex base-pair step parameter mean values with literature. The mean values from the present works simulations (red) are plotted with those from Dans et al, 2012 (gray). Shaded areas represent standard deviations. An absence of the reference line indicates complete overlap.

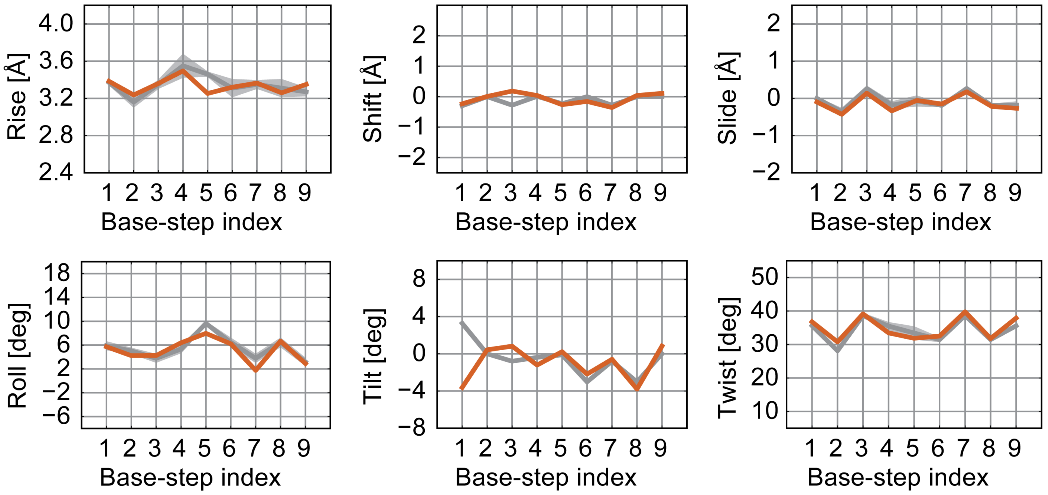

Figure S19: Comparison of the $J24^{1}_{(1)}$ duplex base-pair step parameter mean values with literature. The mean values from the present works simulations (red) are plotted with those from Dans et al, 2012 (gray). Shaded areas represent standard deviations. An absence of the reference line indicates complete overlap.

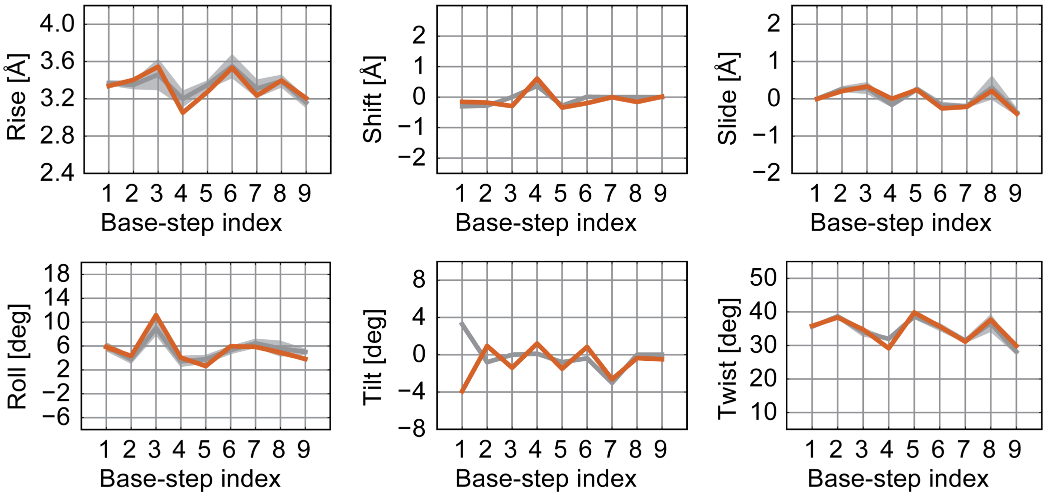

Figure S20: Comparison of the $J24^{1}_{(2)}$ duplex base-pair step parameter mean values with literature. The mean values from the present works simulations (red) are plotted with those from Dans et al, 2012 (gray). Shaded areas represent standard deviations. An absence of the reference line indicates complete overlap.

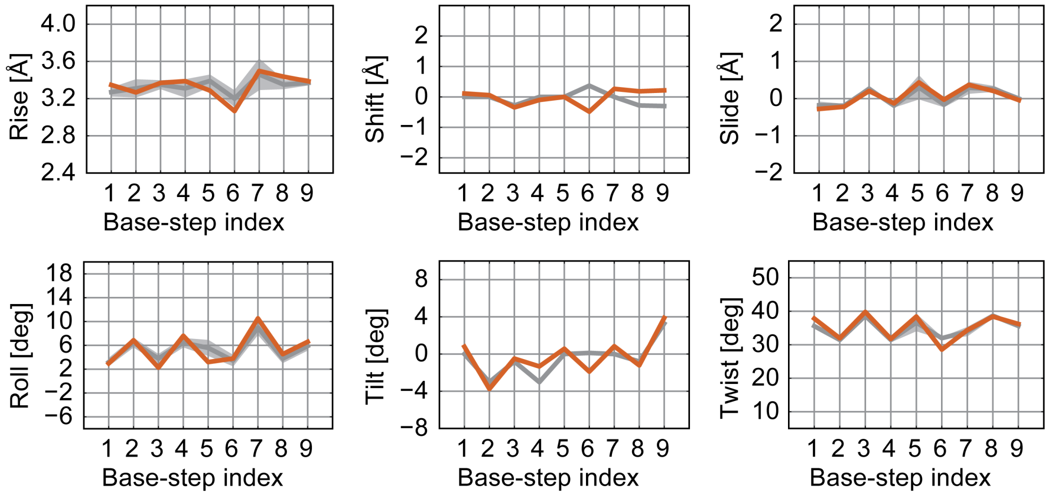

Figure S21: Comparison of the $J24^{2}_{(1)}$ duplex base-pair step parameter mean values with literature. The mean values from the present works simulations (red) are plotted with those from Dans et al, 2012 (gray). Shaded areas represent standard deviations. An absence of the reference line indicates complete overlap.

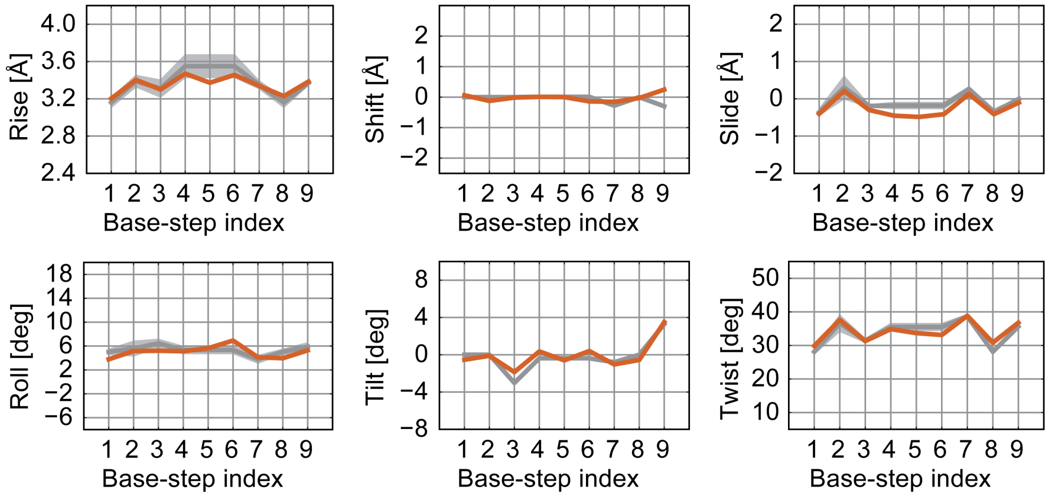

Figure S22: Comparison of the $J24^{2}_{(2)}$ duplex base-pair step parameter mean values with literature. The mean values from the present works simulations (red) are plotted with those from Dans et al, 2012 (gray). Shaded areas represent standard deviations. An absence of the reference line indicates complete overlap.

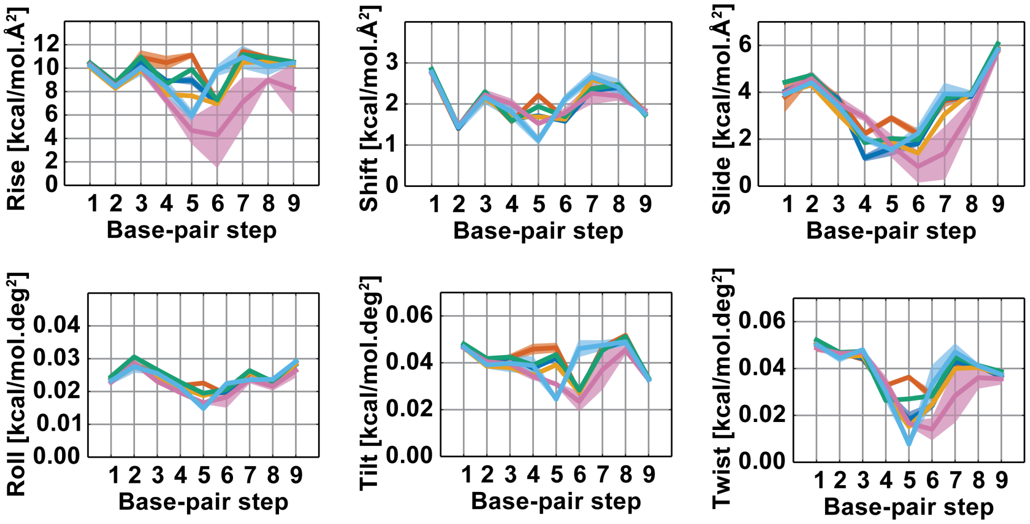

Figure S23: Stiffness constants along the six base-step degrees of freedom for the inner nine base steps of helix 1 are shown for each of the different topologies of J1, as well as for the 4WJ topology of J24, in their isomer 1 forms. The various topologies are color coded as: red (duplex), dark blue (nicked-duplex), orange (SXB), green (SXD), red-purple (J1 4WJ), and light blue (J24 4WJ).

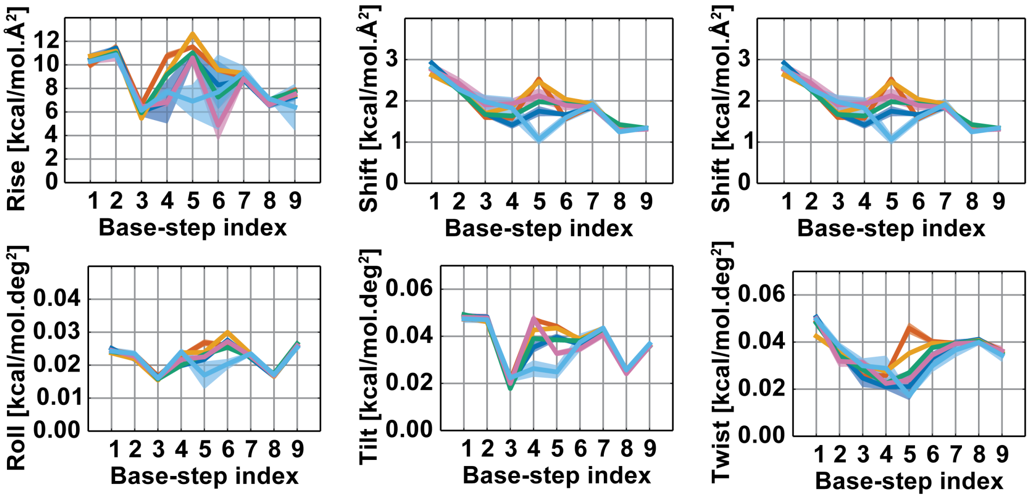

Figure S24: Stiffness constants along the six base-step degrees of freedom for the inner nine base steps of helix 2 are shown for each of the different topologies of J1, as well as for the 4WJ topology of J24, in their isomer 1 forms. The various topologies are color coded as: red (duplex), dark blue (nicked-duplex), orange (SXB), green (SXD), red-purple (J1 4WJ), and light blue (J24 4WJ).

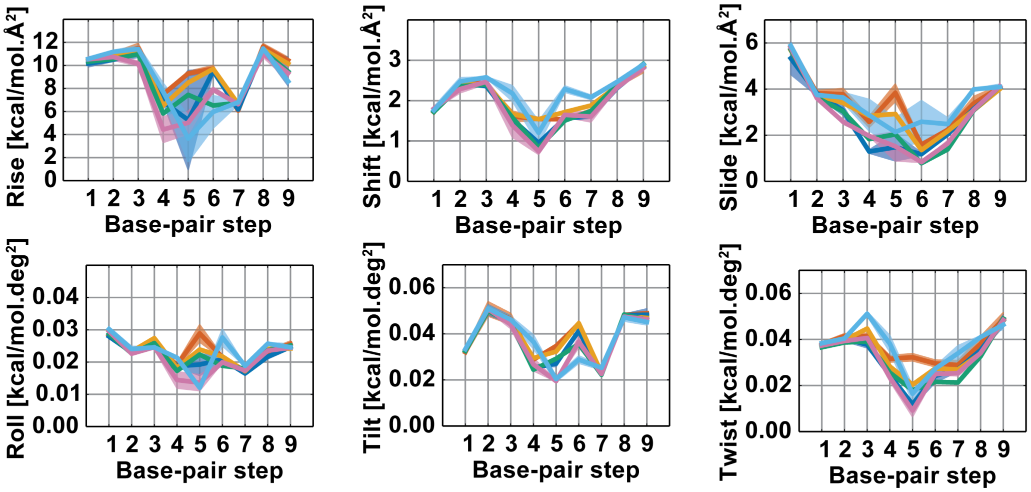

Figure S25: Stiffness constants along the six base-step degrees of freedom for the inner nine base steps of helix 1 are shown for each of the different topologies of J1, as well as for the 4WJ topology of J24, in their isomer 2 forms. The various topologies are color coded as: red (duplex), dark blue (nicked-duplex), orange (SXB), green (SXD), red-purple (J1 4WJ), and light blue (J24 4WJ).

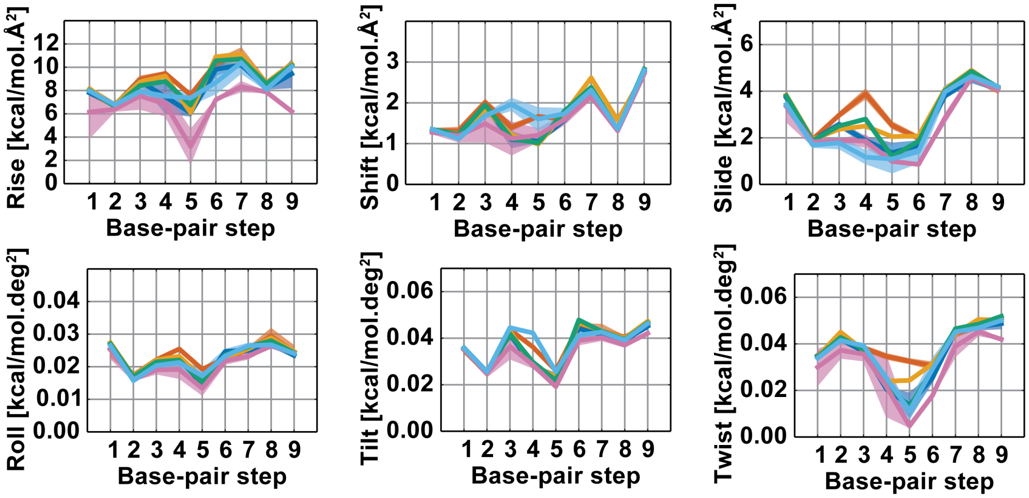

Figure S26: Stiffness constants along the six base-step degrees of freedom for the inner nine base steps of helix 2 are shown for each of the different topologies of J1, as well as for the 4WJ topology of J24, in their isomer 2 forms. The various topologies are color coded as: red (duplex), dark blue (nicked-duplex), orange (SXB), green (SXD), red-purple (J1 4WJ), and light blue (J24 4WJ).

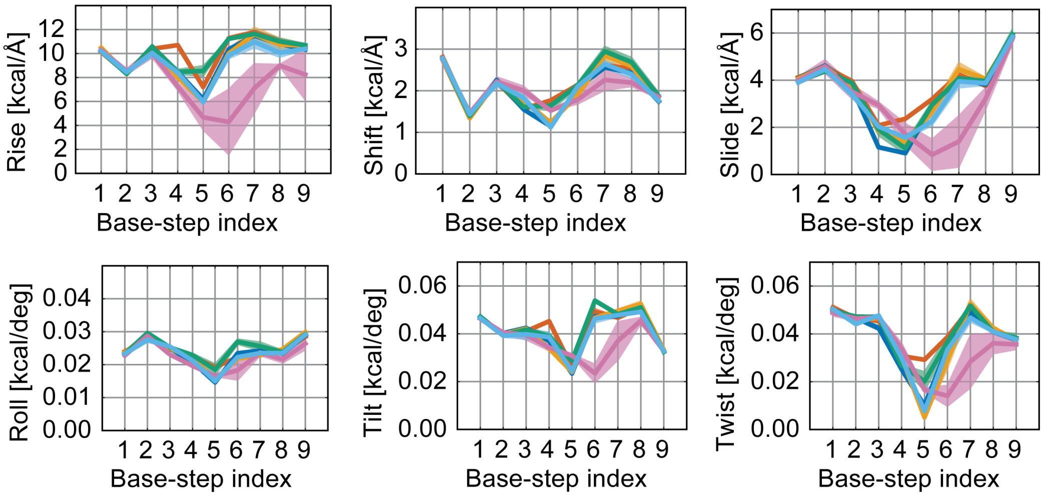

Figure S27: Stiffness constants along the six base-step degrees of freedom for the inner nine base steps of helix 1 are shown for each of the different topologies of J24, as well as for the 4WJ topology of J1, in their isomer 1 forms. The various topologies are color coded as: red (duplex), dark blue (nicked-duplex), orange (SXB), green (SXD), red-purple (J1 4WJ), and light blue (J24 4WJ).

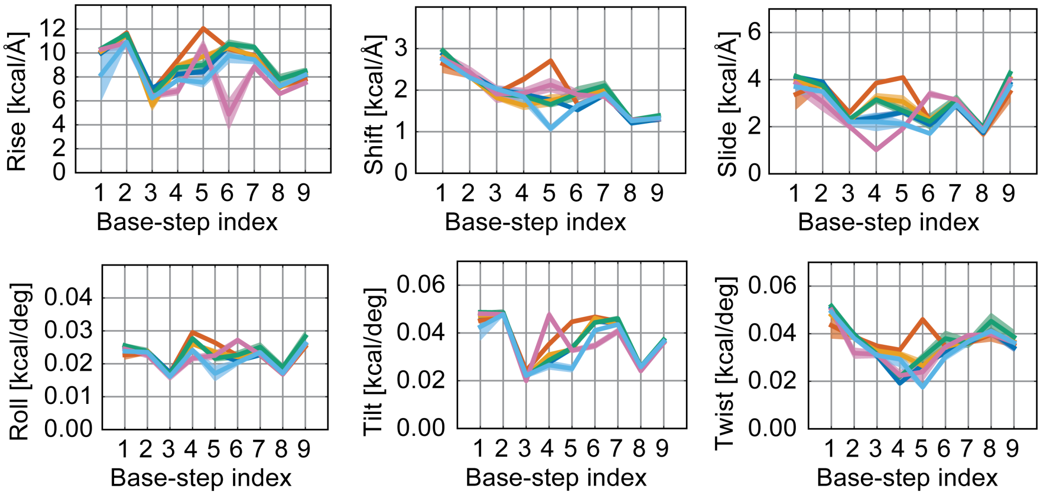

Figure S28: Stiffness constants along the six base-step degrees of freedom for the inner nine base steps of helix 2 are shown for each of the different topologies of J24, as well as for the 4WJ topology of J1, in their isomer 1 forms. The various topologies are color coded as: red (duplex), dark blue (nicked-duplex), orange (SXB), green (SXD), red-purple (J1 4WJ), and light blue (J24 4WJ).

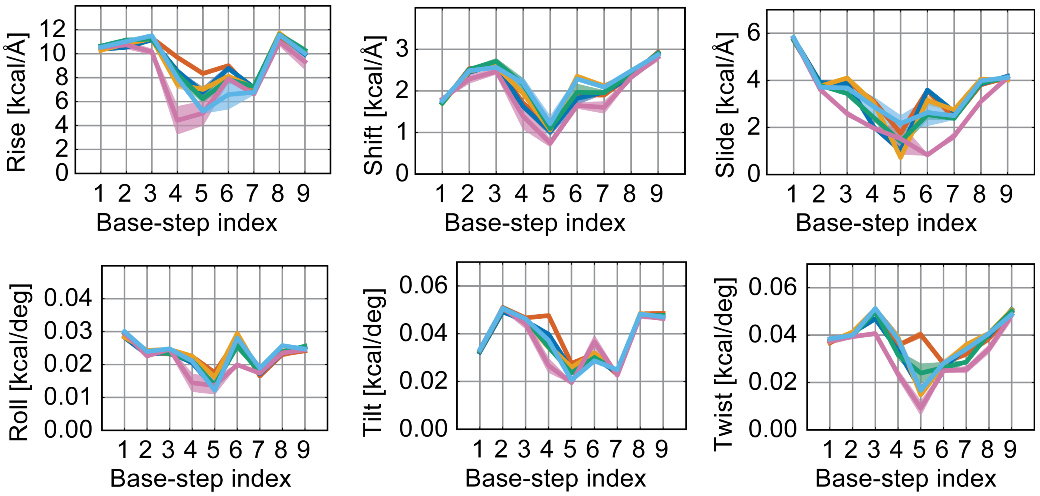

Figure S29: Stiffness constants along the six base-step degrees of freedom for the inner nine base steps of helix 1 are shown for each of the different topologies of J24, as well as for the 4WJ topology of J1, in their isomer 2 forms. The various topologies are color coded as: red (duplex), dark blue (nicked-duplex), orange (SXB), green (SXD), red-purple (J1 4WJ), and light blue (J24 4WJ).

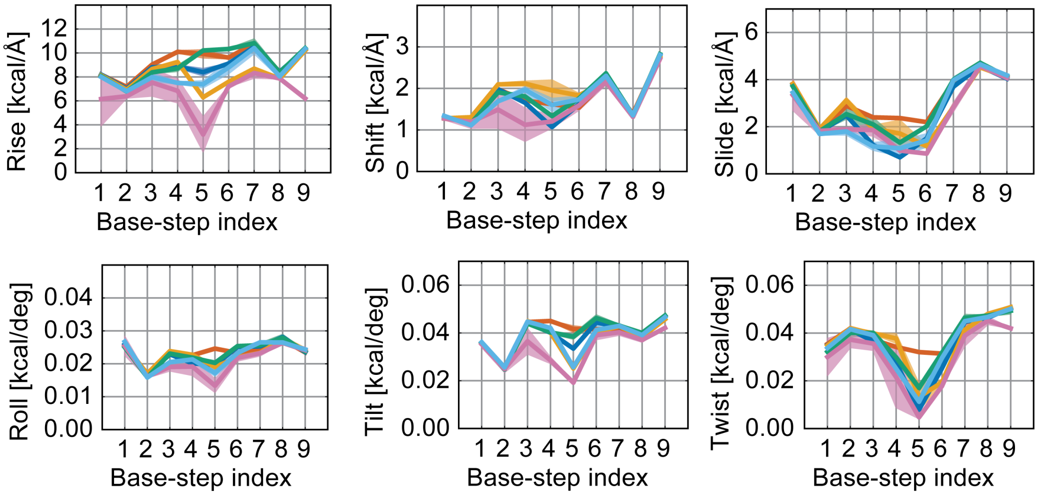

Figure S30: Stiffness constants along the six base-step degrees of freedom for the inner nine base steps of helix 2 are shown for each of the different topologies of J24, as well as for the 4WJ topology of J1, in their isomer 2 forms. The various topologies are color coded as: red (duplex), dark blue (nicked-duplex), orange (SXB), green (SXD), red-purple (J1 4WJ), and light blue (J24 4WJ).

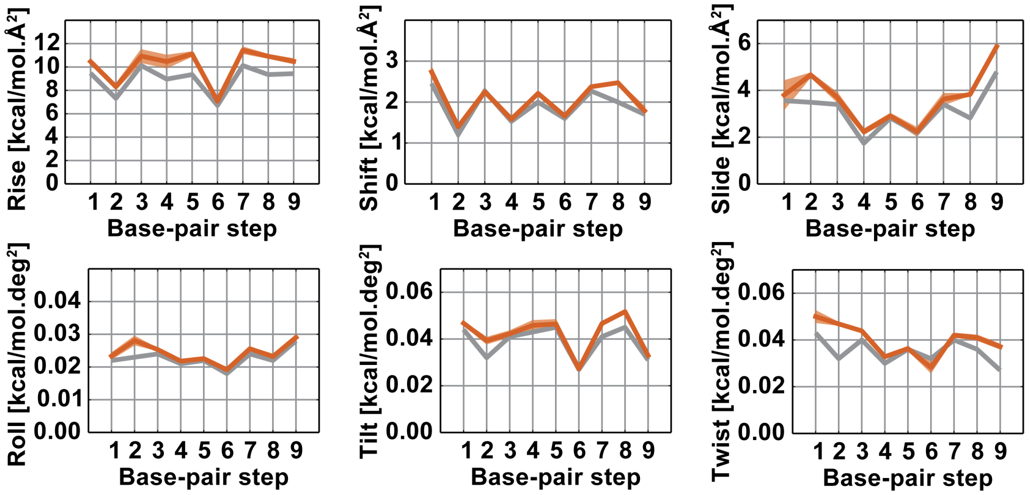

Figure S31: Comparison of the J1$^{1}_{(1)}$ duplex base-pair step diagonal stiffness constants with literature. The mean values from the present works simulations (red) are plotted with those from Pérez et al, 2008 (black). Shaded areas represent standard deviations. An absence of the reference line indicates complete overlap.

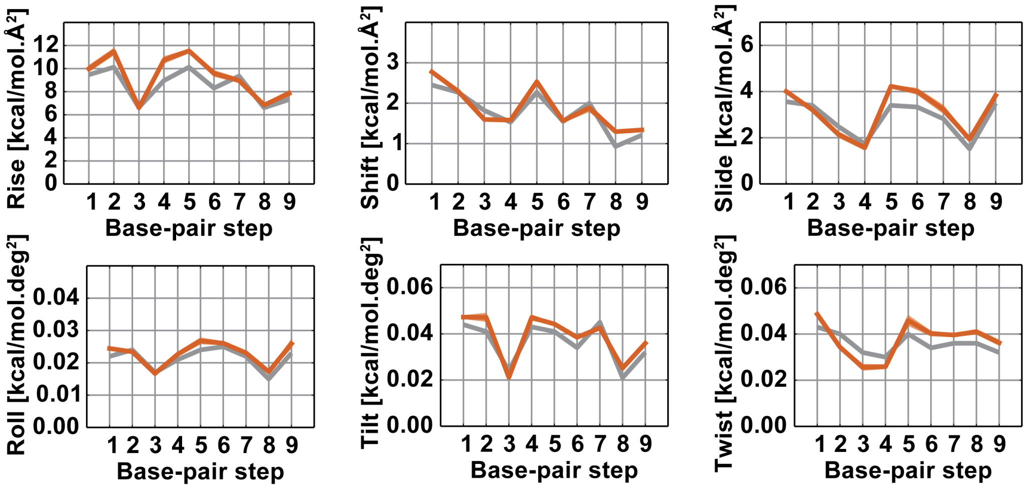

Figure S32: Comparison of the J1$^{1}_{(2)}$ duplex base-pair step diagonal stiffness constants with literature. The mean values from the present works simulations (red) are plotted with those from Pérez et al, 2008 (black). Shaded areas represent standard deviations. An absence of the reference line indicates complete overlap.

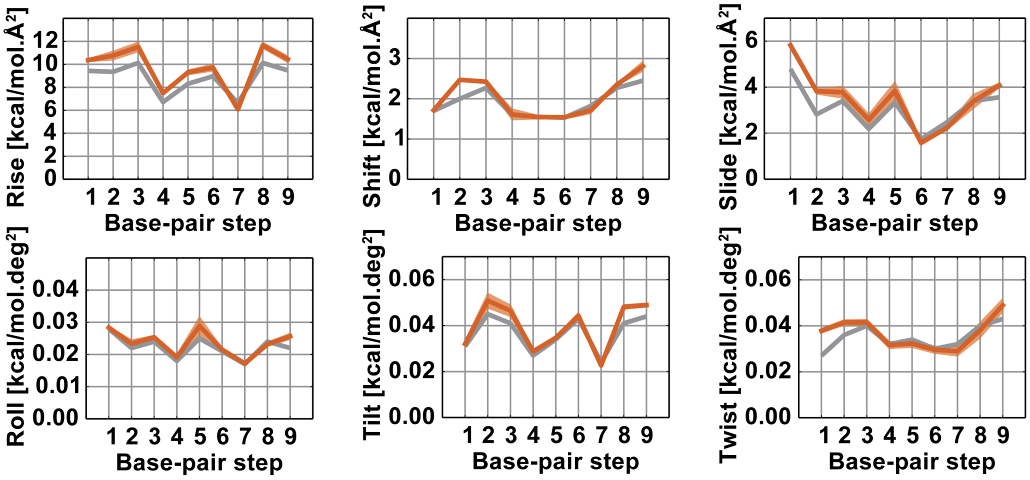

Figure S33: Comparison of the $J1^{2}_{(1)}$ duplex base-pair step diagonal stiffness constants with literature. The mean values from the present works simulations (red) are plotted with those from Pérez et al, 2008 (black). Shaded areas represent standard deviations. An absence of the reference line indicates complete overlap.

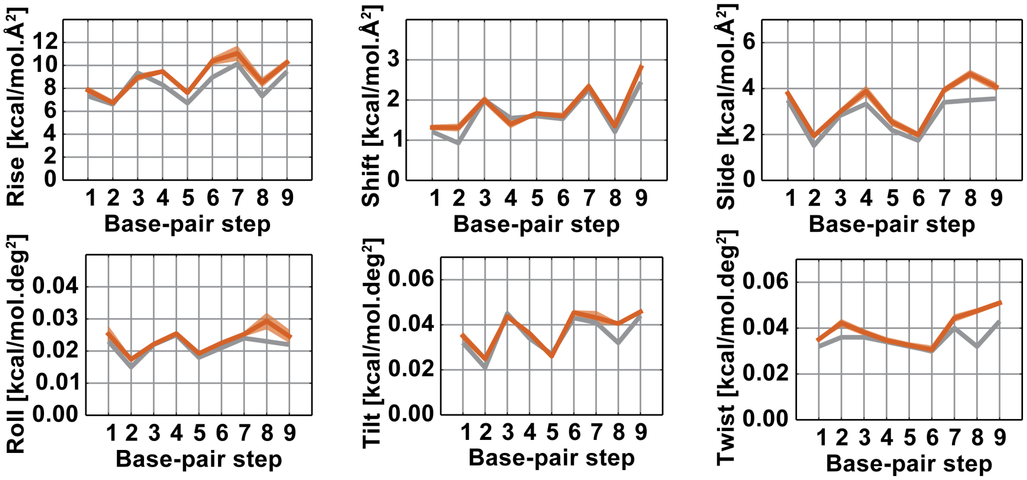

Figure S34: Comparison of the $J1^{2}_{(2)}$ duplex base-pair step diagonal stiffness constants with literature. The mean values from the present works simulations (red) are plotted with those from Pérez et al, 2008 (black). Shaded areas represent standard deviations. An absence of the reference line indicates complete overlap.

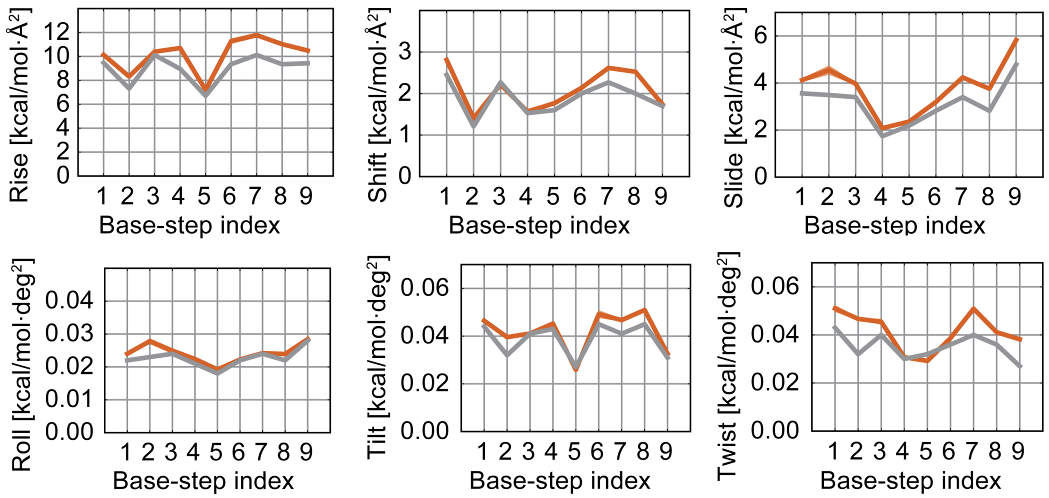

Figure S35: Comparison of the J24$^{1}_{(1)}$ duplex base-pair step diagonal stiffness constants with literature. The mean values from the present works simulations (red) are plotted with those from Pérez et al, 2008 (black). Shaded areas represent standard deviations. An absence of the reference line indicates complete overlap.

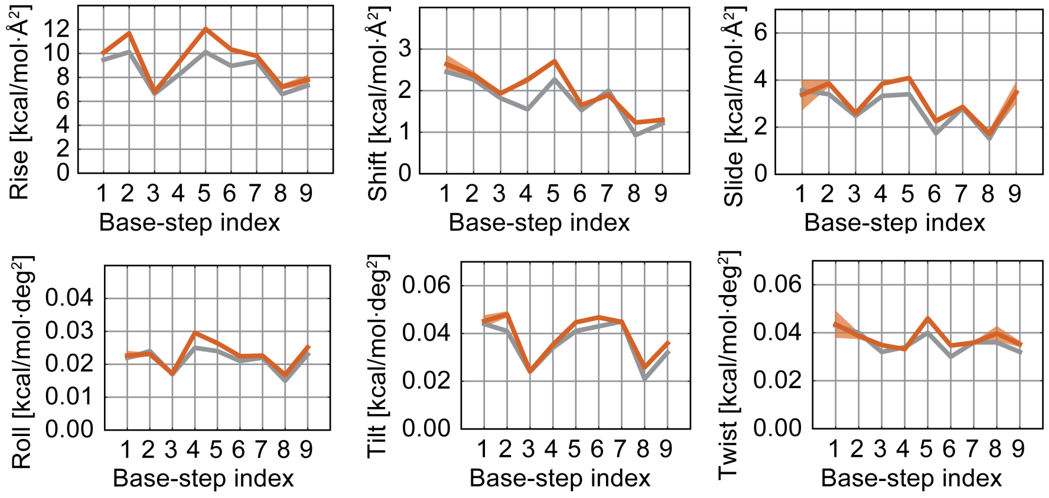

Figure S36: Comparison of the J24$^{1}_{(2)}$ duplex base-pair step diagonal stiffness constants with literature. The mean values from the present works simulations (red) are plotted with those from Pérez et al, 2008 (black). Shaded areas represent standard deviations. An absence of the reference line indicates complete overlap.

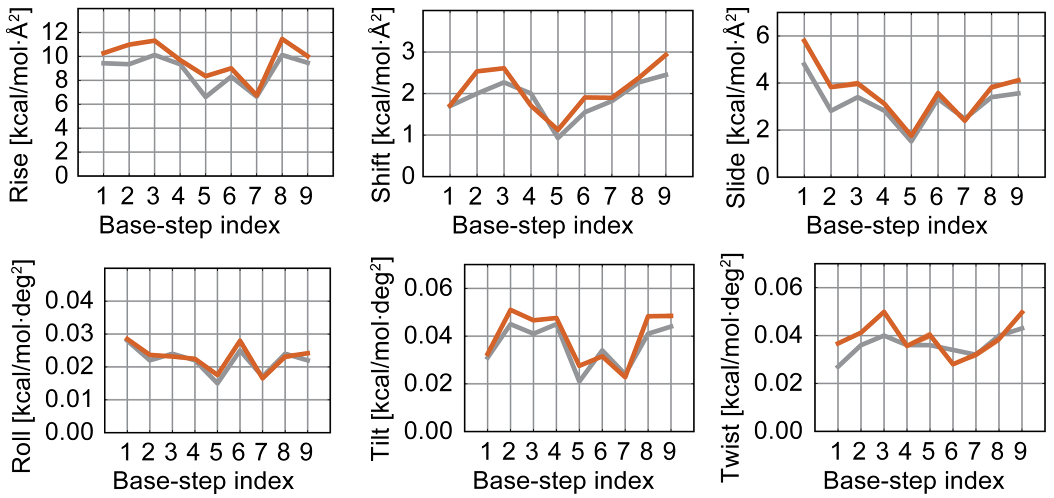

Figure S37: Comparison of the J24$^{2}_{(1)}$ duplex base-pair step diagonal stiffness constants with literature. The mean values from the present works simulations (red) are plotted with those from Pérez et al, 2008 (black). Shaded areas represent standard deviations. An absence of the reference line indicates complete overlap.

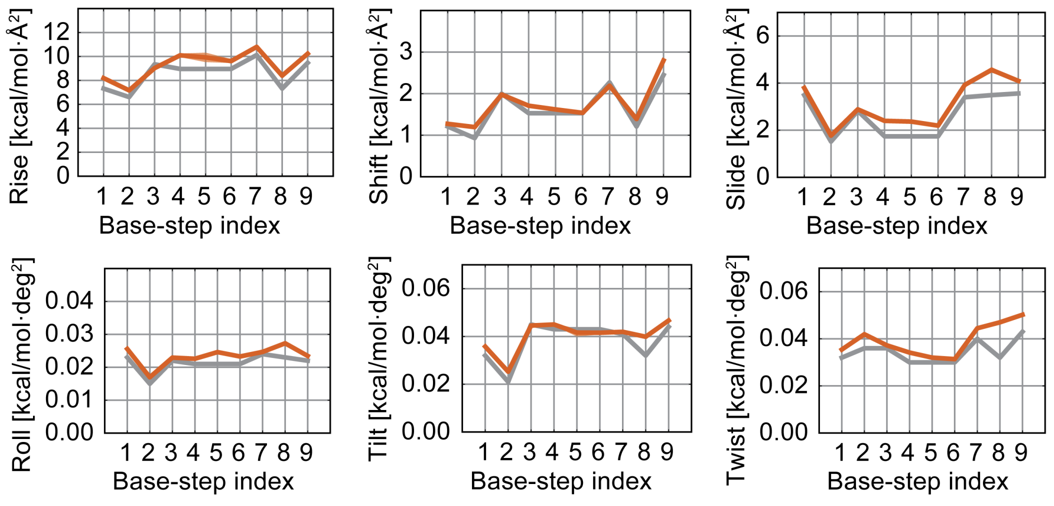

Figure S38: Comparison of the J24$^{2}_{(2)}$ duplex base-pair step diagonal stiffness constants with literature. The mean values from the present works simulations (red) are plotted with those from Pérez et al, 2008 (black). Shaded areas represent standard deviations. An absence of the reference line indicates complete overlap.

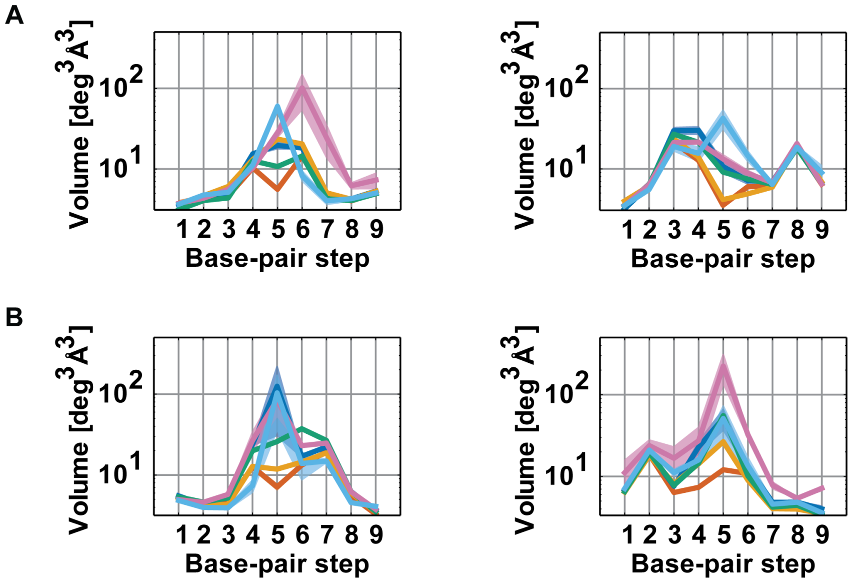

Figure S39: Base-pair step configurational volumes for the different topology systems of J1. The various topologies are color coded as: red (duplex), dark blue (nicked-duplex), orange (SXB), green (SXD), red-purple (J1 4WJ), and light blue (J24 4WJ).

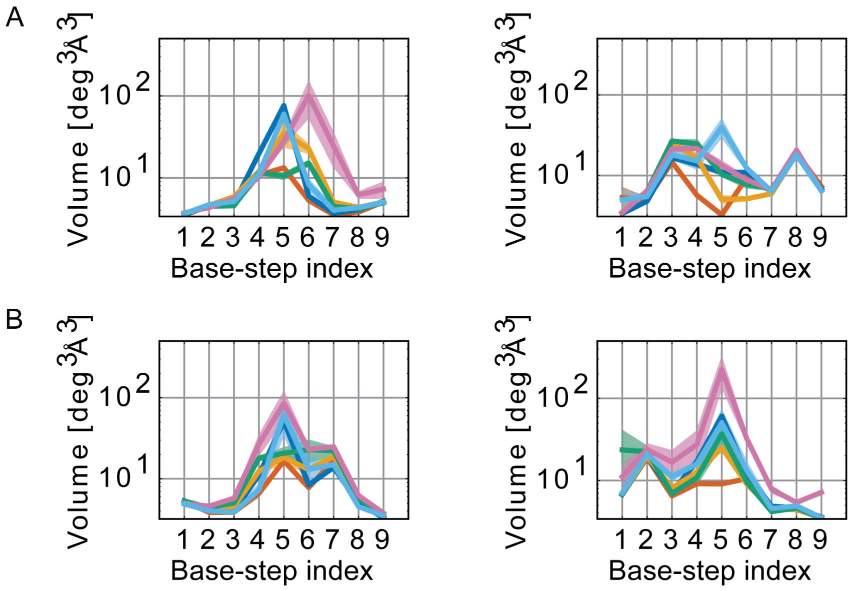

Figure S40: Base-pair step configurational volumes for the different topology systems of J24. The various topologies are color coded as: red (duplex), dark blue (nicked-duplex), orange (SXB), green (SXD), red-purple (J1 4WJ), and light blue (J24 4WJ).

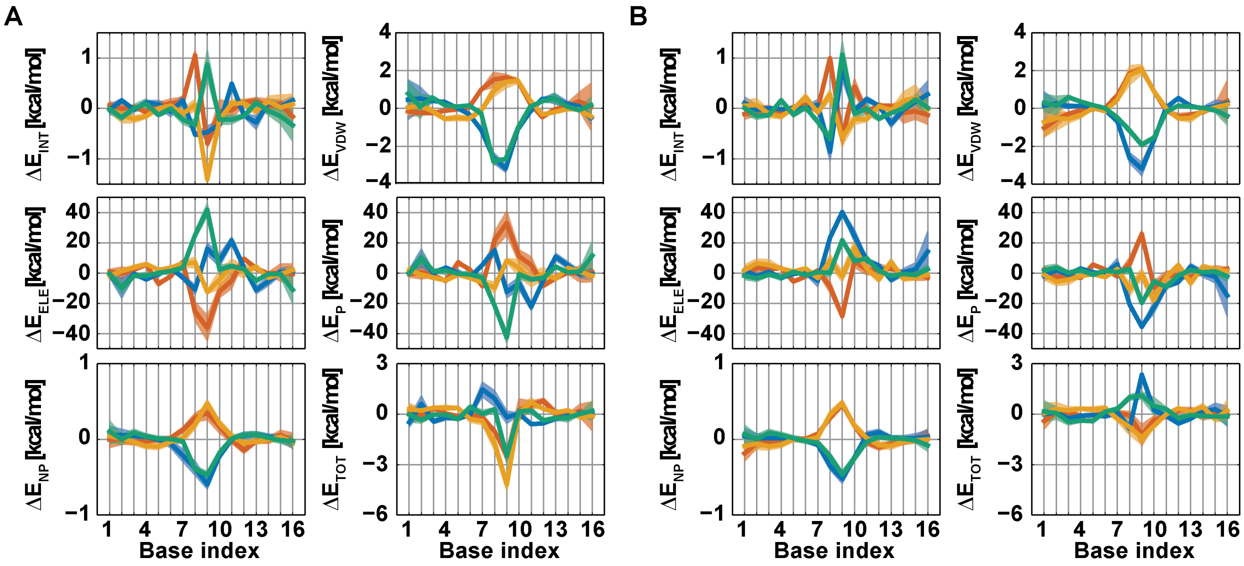

Figure S41: Junction base sequences confer unique free energies. (A) Per-base internal (E_{INT}), van der Waals (E_{VDW}), gas-phase electrostatic (E_{ELE}), polar solvation (E_{P}), non-polar solvation (E_{NP}), and total (E_{TOT}) free energy differences for each chain in the J1 systems and (B) J24 systems. Chains are coloured according to the topology schematics seen in Figure 4 and the shaded regions represent the standard deviations across replicas.

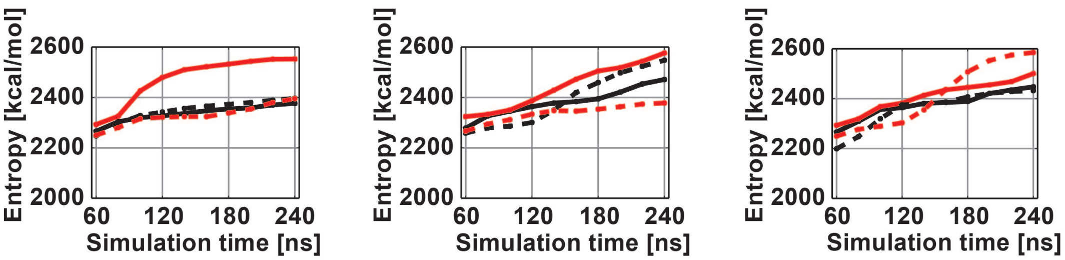

Figure S42: Solute entropies for the J1 and J24 junction systems. The entropies for replica 1 (left), replica 2 (middle), and replica 3 (right) are shown for $J1^{1}$ (solid black), $J1^{2}$ (solid red), $J24^{1}$ (dashed black), and $J24^{2}$ (dashed red) are shown at various trajectory lengths. Each nanosecond corresponds to 1,000 structures.

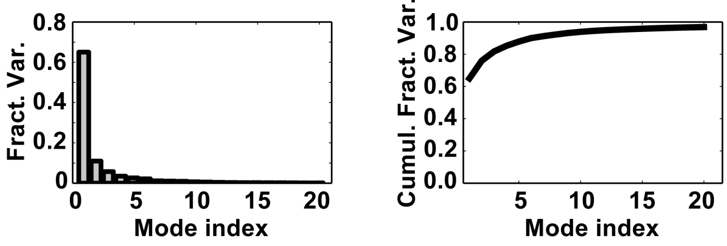

Figure S43: The fractional variance (left) and cumulative fractional variance (right) for the 20 highest variance meta-ensemble modes, ranked by eigenvalue.

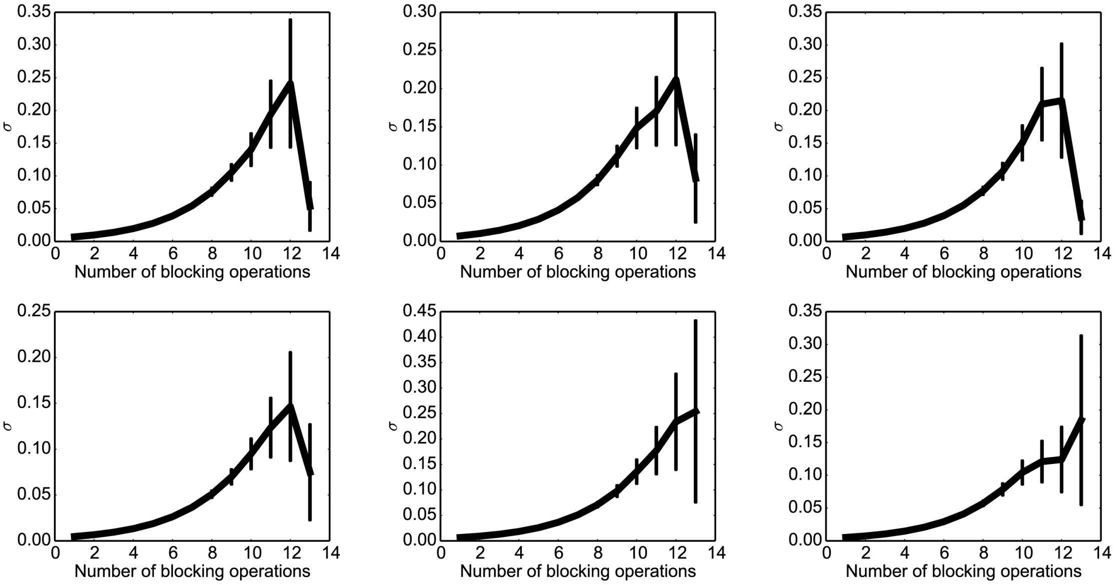

Figure S44: Blocking curves for the J1 system replicates. Blocking curves are shown for each of the three replicates of J1[1] (top row) and J1[2] (bottom row), performed on projections of the individual trajectories onto the second junction essential mode (J_{roll}). The block size is determined as 2^n ps, where n is the number of blocking operations and each.

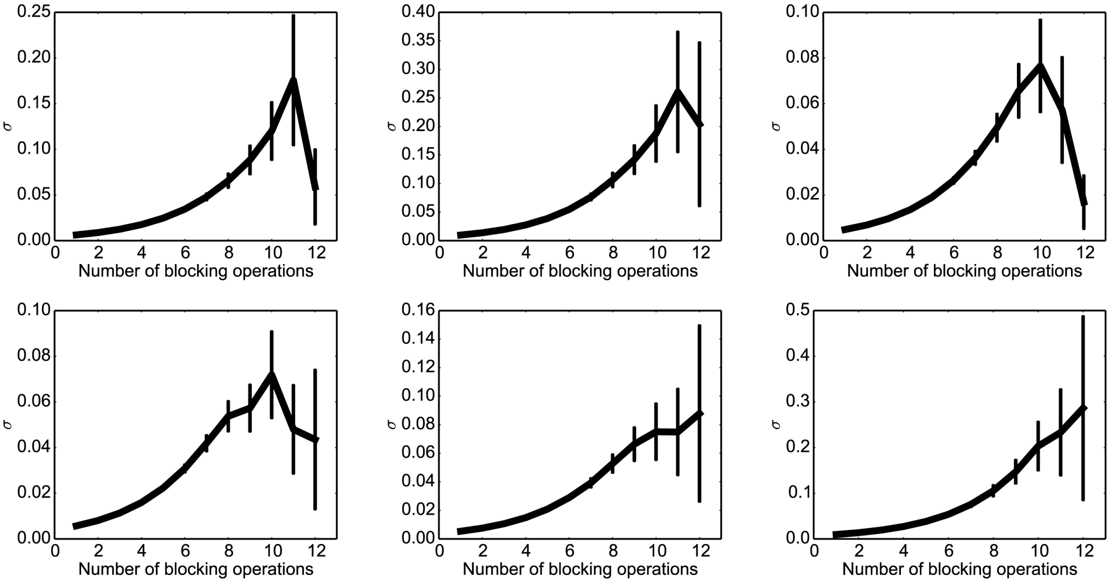

Figure S45: Blocking curves for the J24 system replicates. Blocking curves are shown for each of the three replicates of J24[1] (top row) and J24[2] (bottom row), performed on projections of the individual trajectories onto the second junction essential mode (J_{roll}). The block size is determined as 2^n ps, where n is the number of blocking operations.

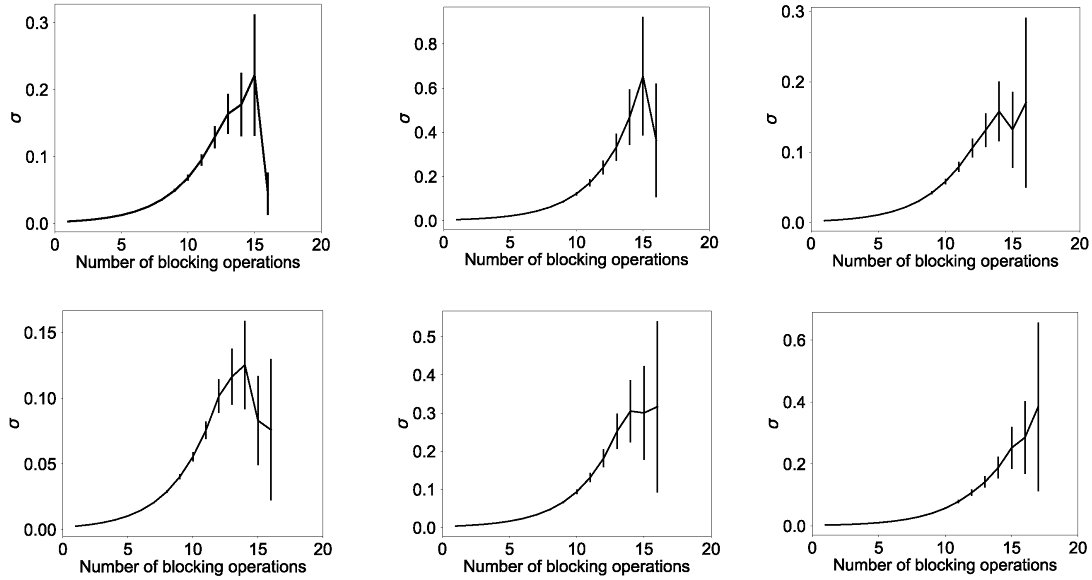

Figure S46: Blocking curves for the J1 system replicates. Blocking curves are shown for each of the three replicates of $J1^1$ (top row) and $J1^2$ (bottom row), performed on projections of the individual trajectories onto the first junction essential mode (J_{twist}). The block size is determined as 2^n ps, where n is the number of blocking operations and each.

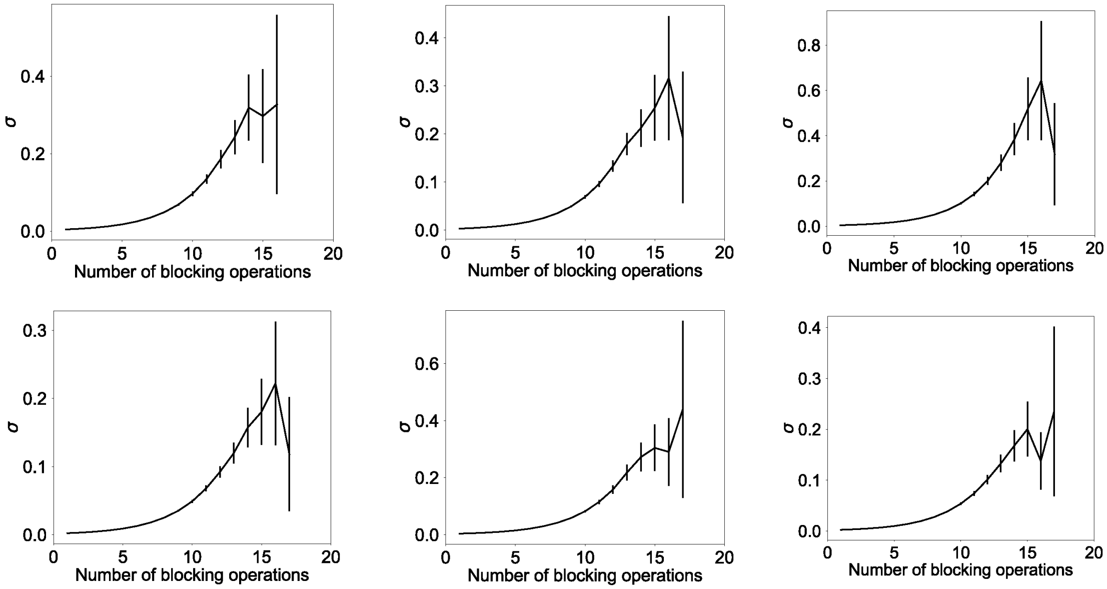

Figure S47: Blocking curves for the J24 system replicates. Blocking curves are shown for each of the three replicates of $J24^1$ (top row) and $J24^2$ (bottom row), performed on projections of the individual trajectories onto the first junction essential mode (J_{twist}). The block size is determined as 2^n ps, where n is the number of blocking operations.
